# Supplementary material for: Rapid complete reconfiguration induced actual active species for industrial hydrogen evolution reaction
Source: Nat Commun. 2022 Oct 2;13:5785. doi: 10.1038/s41467-022-33590-5 (PMC9527236; doi:10.1038/s41467-022-33590-5)
Supplement: Supplementary file 1 — Supplementary Information [file 41467_2022_33590_MOESM1_ESM.pdf]

Supporting Information

Rapid complete reconfiguration induced actual active species for industrial hydrogen evolution reaction

Luqi Wang[1], Yixin Hao[1], Liming Deng[1], Feng Hu[1], Sheng Zhao[1], Linlin Li[1], Shengjie Peng[*1]

[1]Jiangsu Key Laboratory of Electrochemical Energy Storage Technologies, College of Materials Science and Technology, Nanjing University of Aeronautics and Astronautics, Nanjing 210016, China.

Email: pengshengjie@nuaa.edu.cn

Supplementary Figures

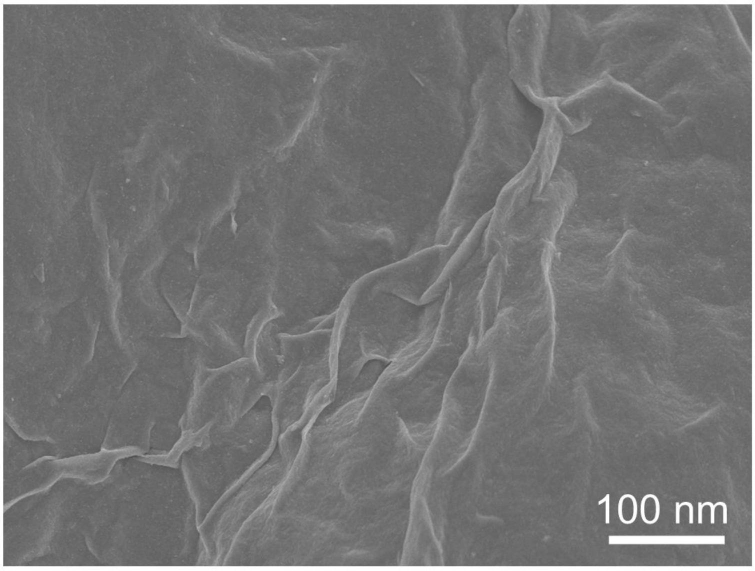

Supplementary Fig. 1. Morphology characterization. SEM image of exfoliated ultrathin MXene nanosheets.

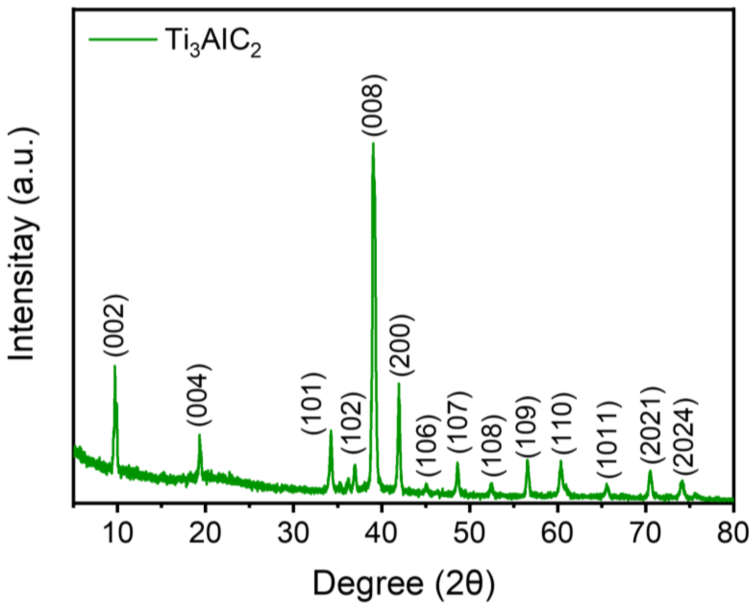

Supplementary Fig. 2. Phase characterization. XRD pattern of Ti_3AlC_2.

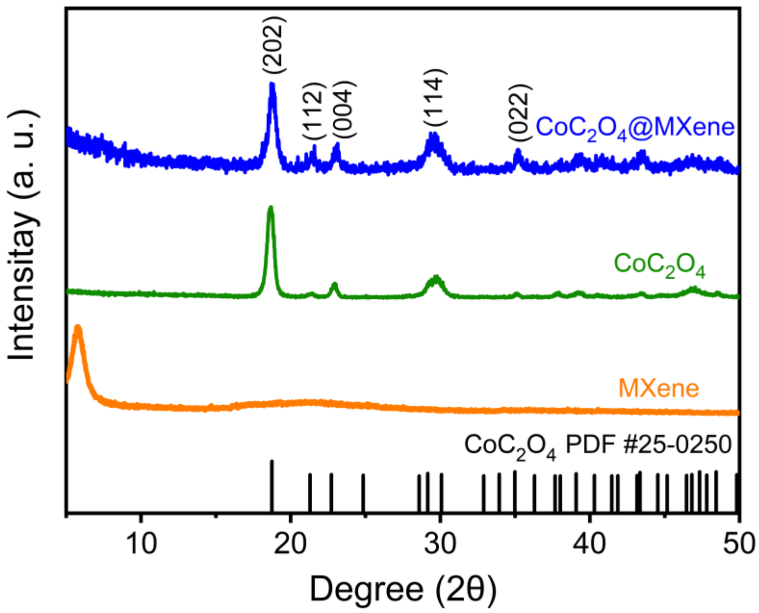

Supplementary Fig. 3. Phase characterization. XRD patterns of MXene, CoC_2O_4, and CoC_2O_4@MXene, respectively.

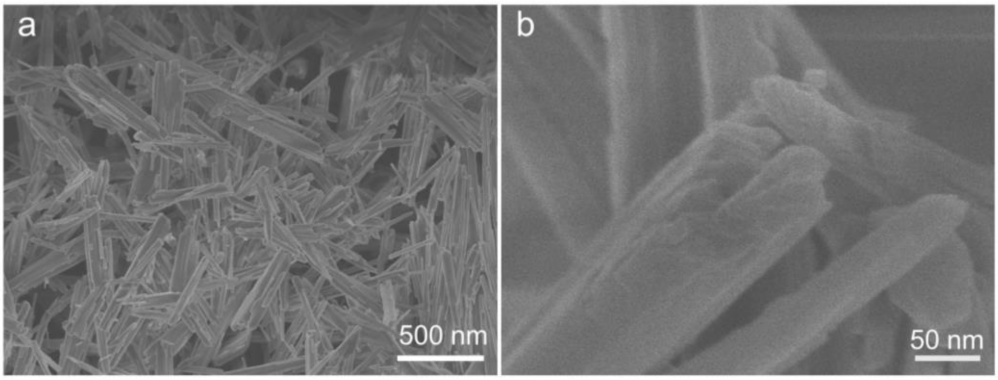

Supplementary Fig. 4. Morphology characterization. a) Low-magnified and b) high-magnified SEM images of CoC_2O_4, respectively.

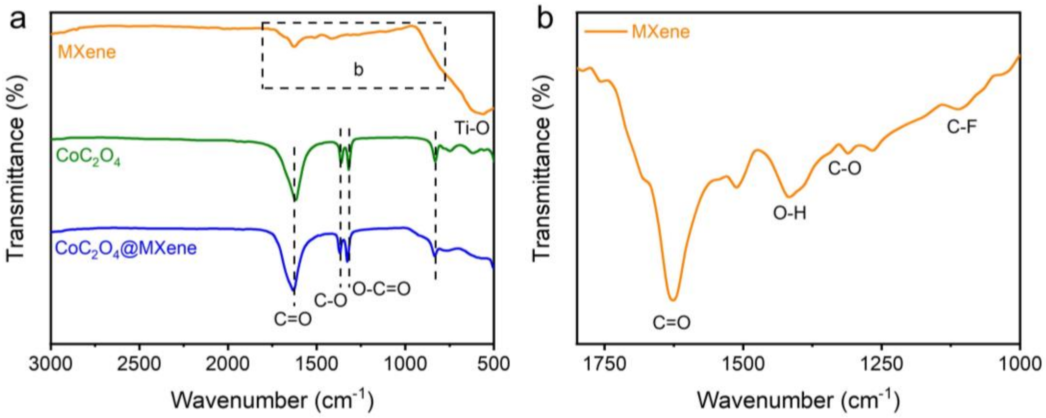

Supplementary Fig. 5. Surface functional group characterization. a) FTIR spectra of MXene, CoC_2O_4, and CoC_2O_4@MXene, respectively. b) Enlarged FTIR spectrum of MXene.

The absorption bands of the FTIR spectra of MXene at 568.28, 1110.63, and 1417.35 cm^{-1} correspond to the stretching vibrations of the -O, -F, and -OH functional groups on the surface, respectively. This further verifies the species of MXene surface functional groups.

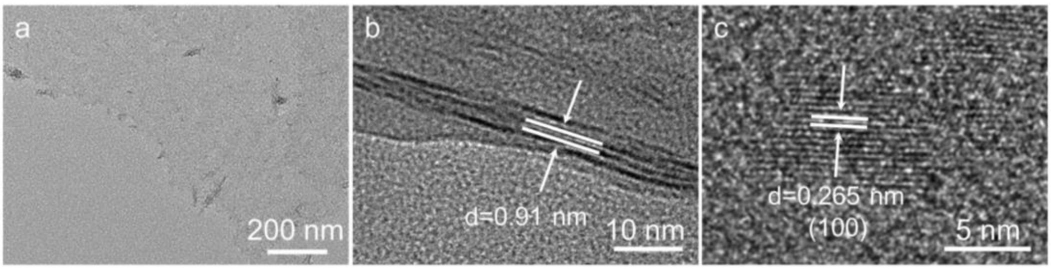

Supplementary Fig. 6. Morphology characterization. a) TEM and b, c) HRTEM images of MXene.

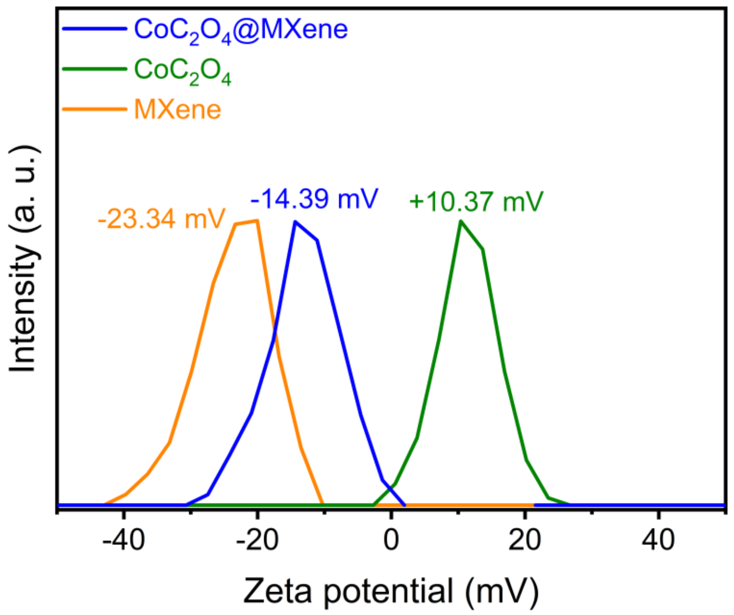

Supplementary Fig. 7. Surface electronegativity characterization. Zeta spectra of MXene, CoC_2O_4, and CoC_2O_4@MXene, respectively.

Due to the presence of surface functional groups such as -O, -F, and -OH, the MXene nanosheets are endowed with a negative charge, which is also demonstrated by the zeta potential of -23.34 mV. After the combination of CoC_2O_4 with MXene, the zeta potential of CoC_2O_4@MXene (+14.39 mV) is positively shifted compared with MXene. This is ascribed to Coulombic interactions between MXene and the positively charged CoC_2O_4, which screens the surface charges of MXene.

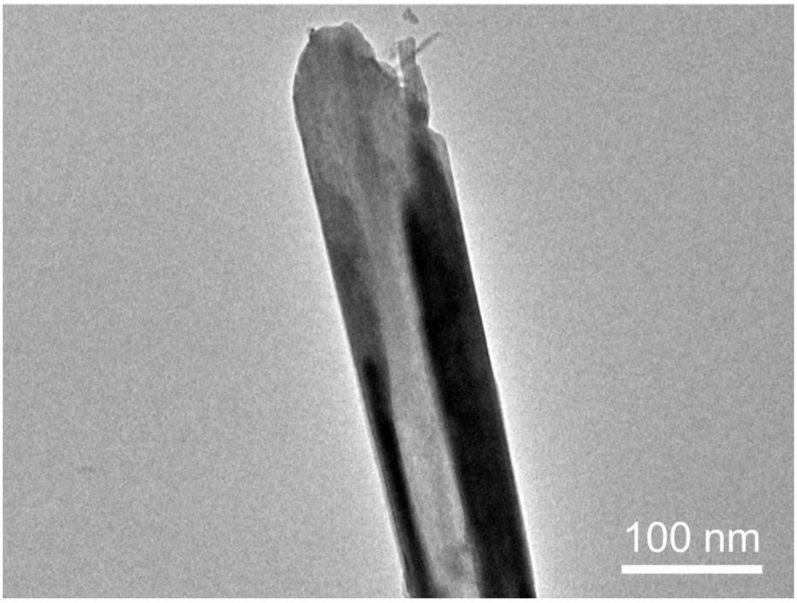

Supplementary Fig. 8. Morphology characterization. TEM image of CoC_2O_4@MXene.

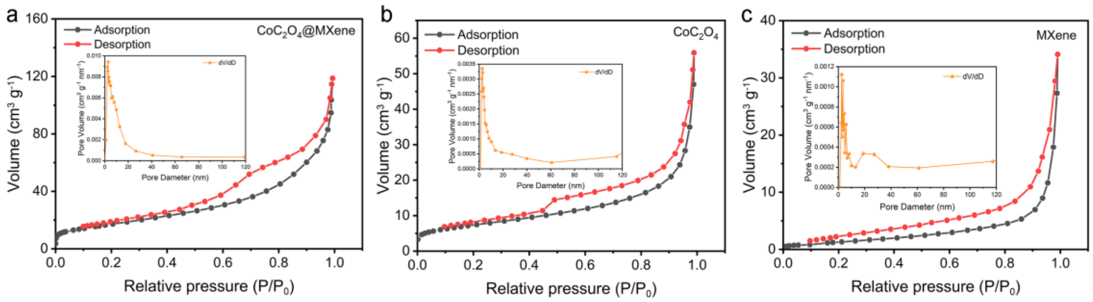

Supplementary Fig. 9. Specific surface area and pore size characterization. The N_2 absorption and desorption isotherms of a) CoC_2O_4@MXene, b) CoC_2O_4, and c) MXene (inset: pore size distribution). The BET values of CoC_2O_4@MXene, CoC_2O_4, and MXene are 62.8, 26.4, and 5.4 m^2 g^{-1}, respectively.

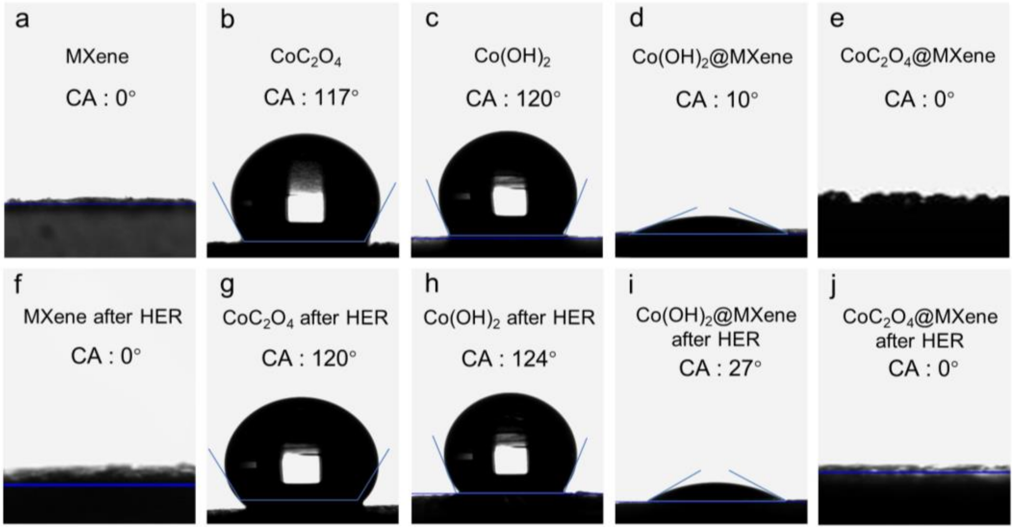

Supplementary Fig. 10. Hydrophilic and hydrophobic characterization. a-e) Contact angles of water droplets on MXene, CoC_2O_4, $Co(OH)_2$, $Co(OH)_2$@MXene, and CoC_2O_4@MXene, respectively. f-j) Contact angles of water droplets on MXene, CoC_2O_4, $Co(OH)_2$, $Co(OH)_2$@MXene, and CoC_2O_4@MXene after HER, respectively.

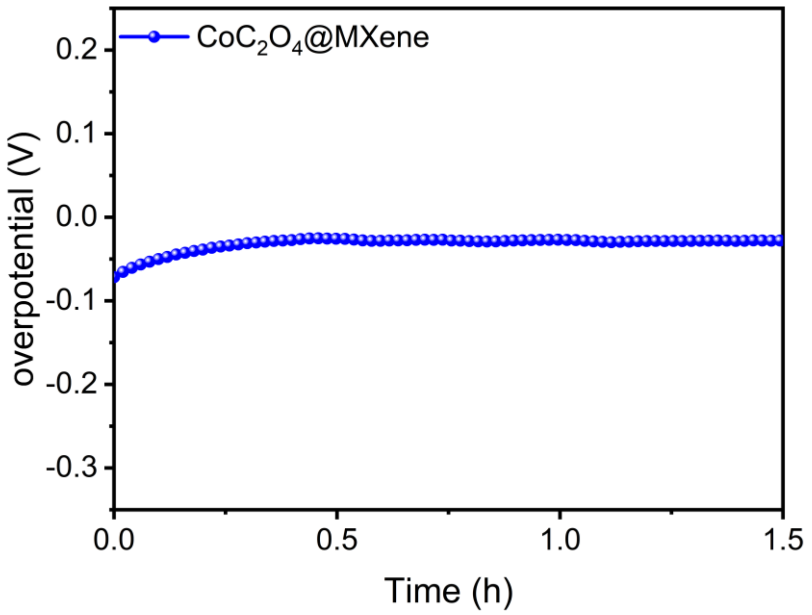

Supplementary Fig. 11. Activation process stability test. Chronopotentiometry curves of CoC_2O_4@MXene at constant current densities of 10 mA cm^{-2}.

As shown in Supplementary Fig. 11, the catalyst can maintain 28 mV in the 1 h chronopotentiometry test after a short activation process. Therefore, the catalyst remained stable without further reconstruction at 28 mV.

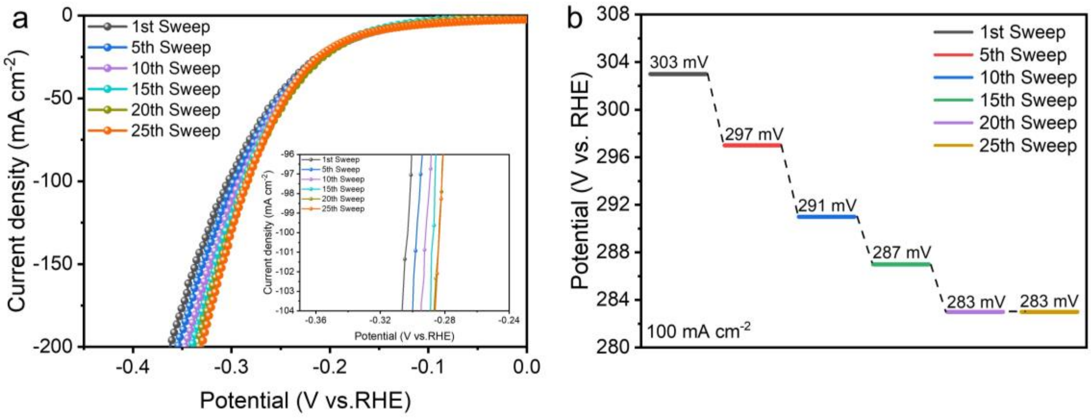

Supplementary Fig. 12. Activation process LSV cycle. a) Polarization curves and b) Corresponding overpotentials (@100 mA cm^{-2}) of CoC_2O_4 in 1.0 M KOH.

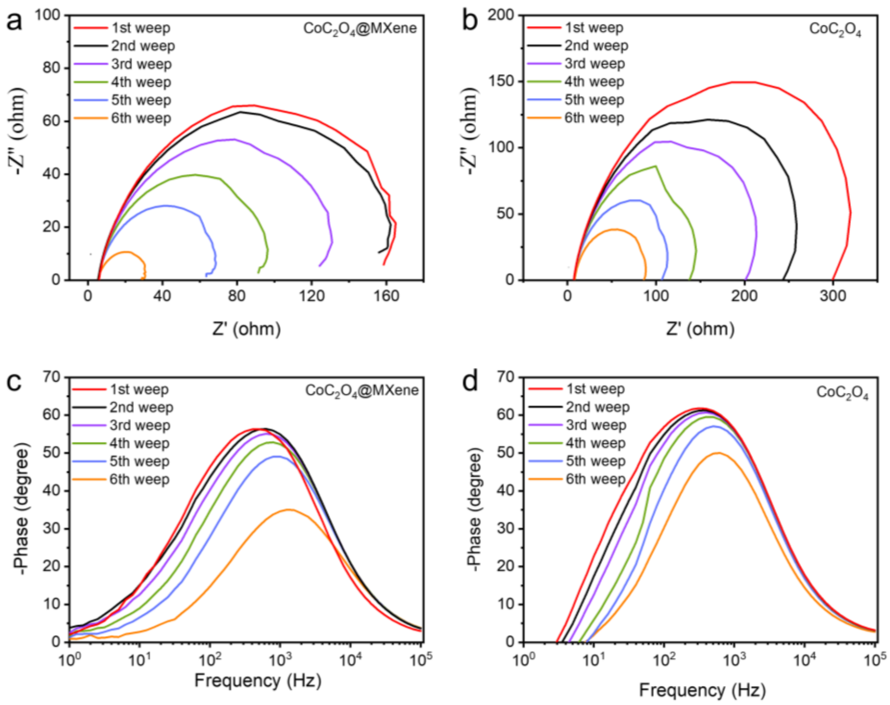

Supplementary Fig. 13. Impedance and Bode Plot analysis. a, b) EIS and c, d) Bode plots of different sweep cycles for CoC_2O_4@MXene and CoC_2O_4, respectively.

To further explore the effect of MXene on the reconfiguration process, the mass and charge transfer processes are investigated using rotating disk electrodes. CoC_2O_4@MXene exhibits smaller internal resistance and higher surface mass accumulation with the increased cycle number compared with CoC_2O_4.

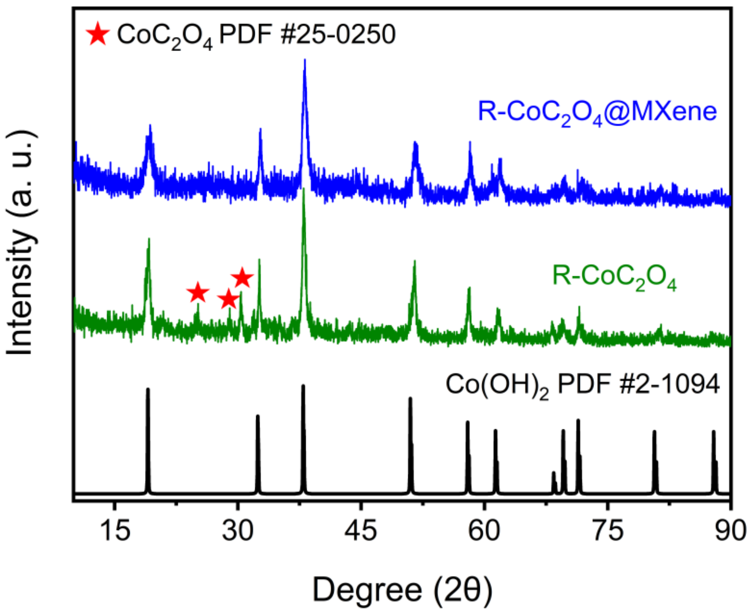

Supplementary Fig. 14. Phase characterization. XRD patterns of R-CoC_2O_4@MXene and R-CoC_2O_4, respectively.

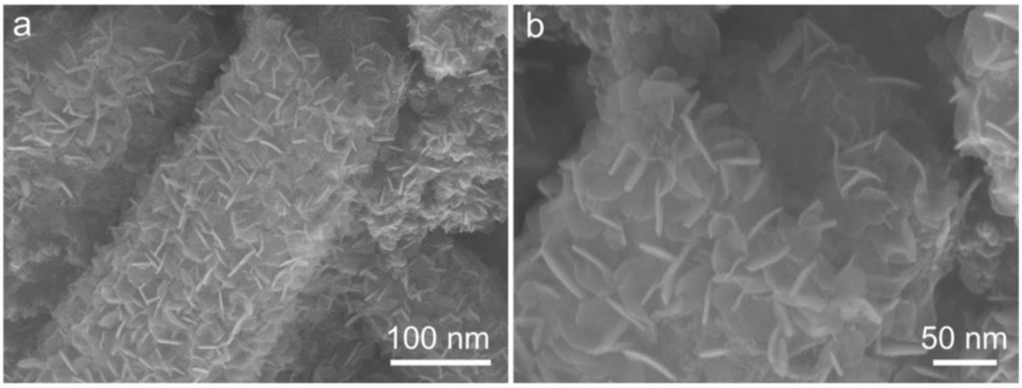

Supplementary Fig. 15. Morphology characterization. a) Low-magnified and b) high-magnified SEM images of R-CoC_2O_4@MXene after cycling, respectively.

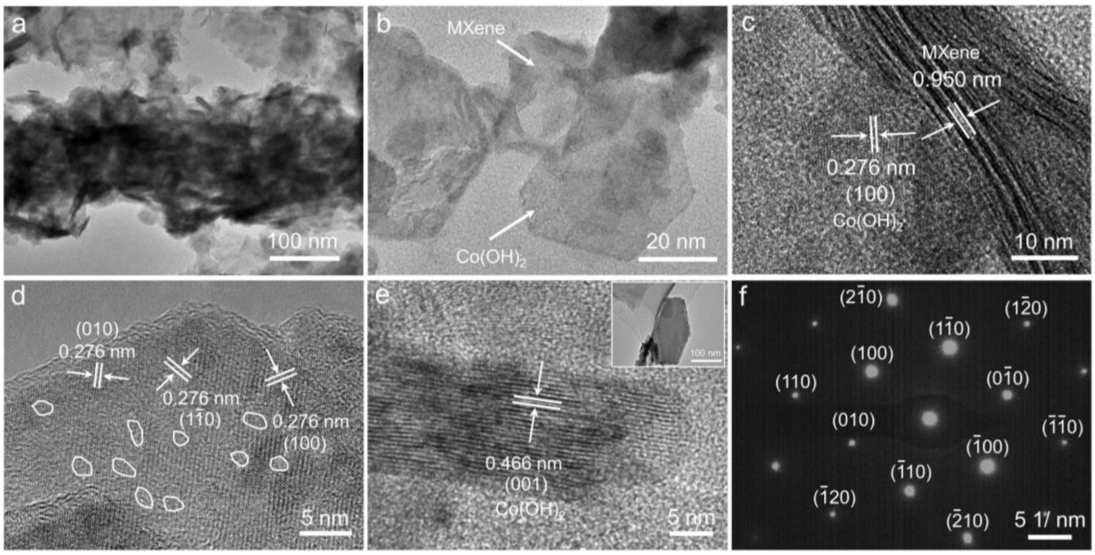

Supplementary Fig. 16. Morphology and structural characterization. a, b) Low-magnified images, c-e) high-magnified TEM images, and f) SAED pattern of the R-CoC_2O_4@MXene after cycling, respectively.

Supplementary Fig. 16d shows HRTEM image of $Co(OH)_2$ hexagonal nanosheet, in which the three types of lattice fringes correspond to interplanar spacings of 0.276 nm. The angles between any of them are 60°. These planes are consistent with the SAED pattern projection of $Co(OH)_2$ along the [001] direction (Supplementary Fig. 16f and 20e). According to the crystal structure of $Co(OH)_2$, the $(\bar{1}10)$, (010), and (100) planes are equivalent. Therefore, the (001) and (100) planes are exposed on the hexagonal $Co(OH)_2$ nanosheets (Supplementary Fig. 20f). Moreover, Supplementary Fig. 16e shows that the side-view HRTEM images of $Co(OH)_2$ nanosheet can clearly present well-resolved lattice fringes. The interlayer spacing of 0.466 nm corresponds to the (001) plane of $Co(OH)_2$.

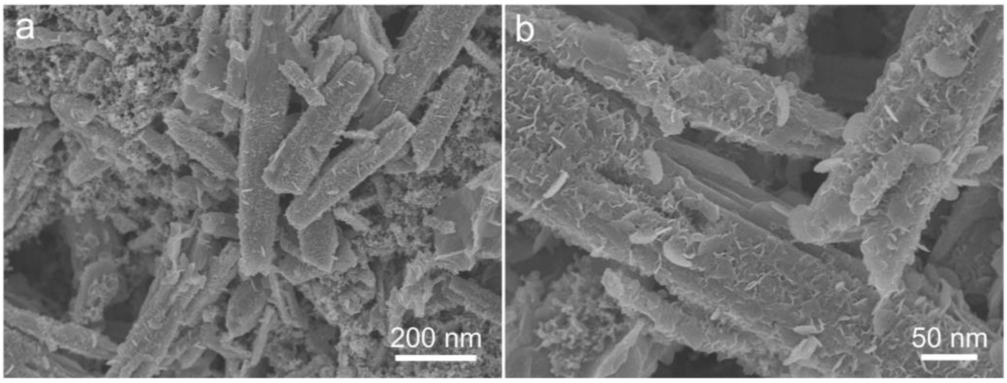

Supplementary Fig. 17. Morphology characterization. a) Low-magnified and b) high-magnified SEM images of R-CoC_2O_4 after cycling, respectively.

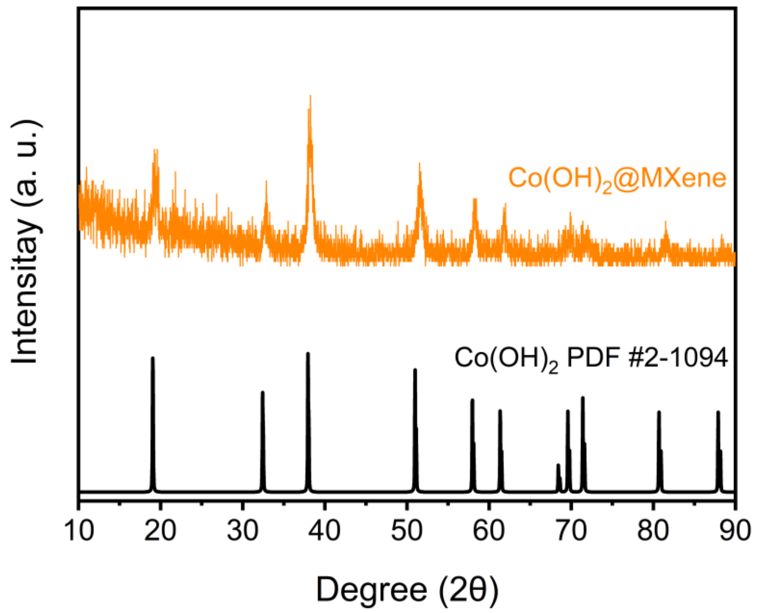

Supplementary Fig. 18. Phase characterization. XRD pattern of as-prepared $Co(OH)_2$@MXene.

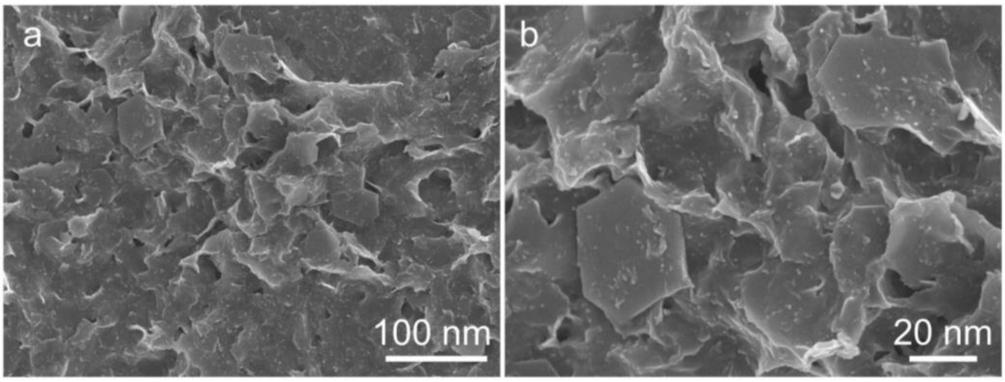

Supplementary Fig. 19. Morphology characterization. a) Low-magnified and b) high-magnified SEM images of as-prepared $Co(OH)_2$@MXene, respectively.

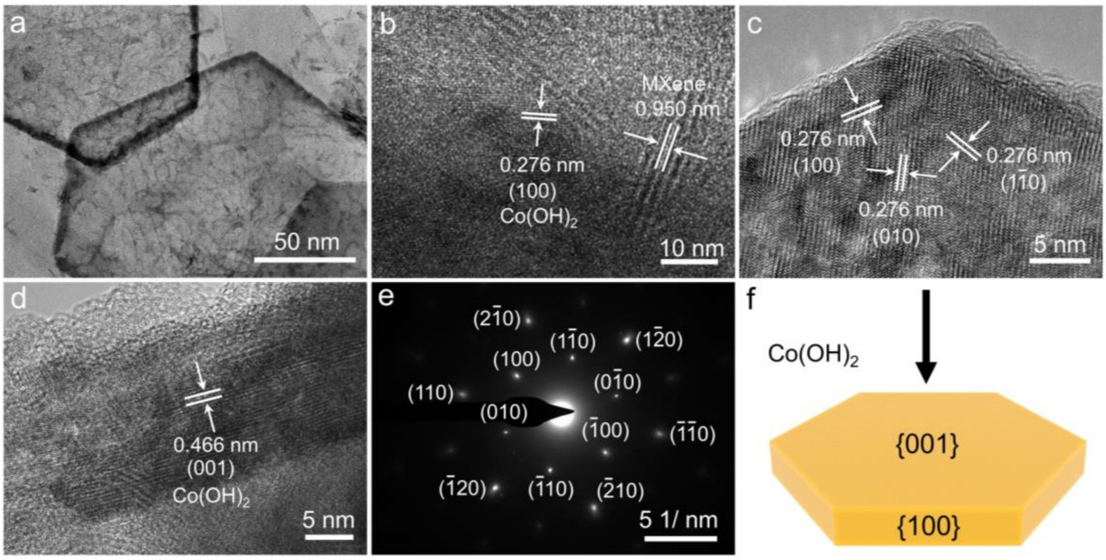

Supplementary Fig. 20. Morphology and structural characterization. a) Low-magnified image, b-d) high-magnified TEM images, and e) SAED pattern of the as-prepared $Co(OH)_2$@MXene, respectively. f) Simulated crystal morphology of $Co(OH)_2$.

As the growth direction of $Co(OH)_2$ nanosheets belongs to the two-dimensional extension type, the growth rate is faster in the <100> direction. Therefore, the $Co(OH)_2$ in R-CoC_2O_4@MXene and as-prepared $Co(OH)_2$@MXene grows along the <100> direction and is mainly exposed to {001} planes.

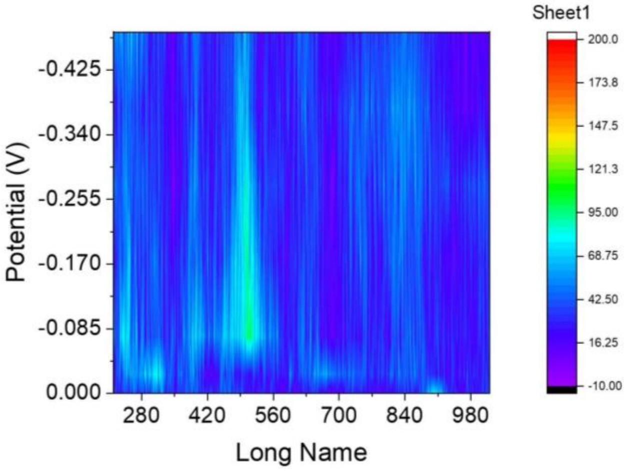

Supplementary Fig. 21. **In situ Raman characterization of CoC_2O_4@MXene.** Potential-dependent in situ Raman spectra of R-CoC_2O_4@MXene.

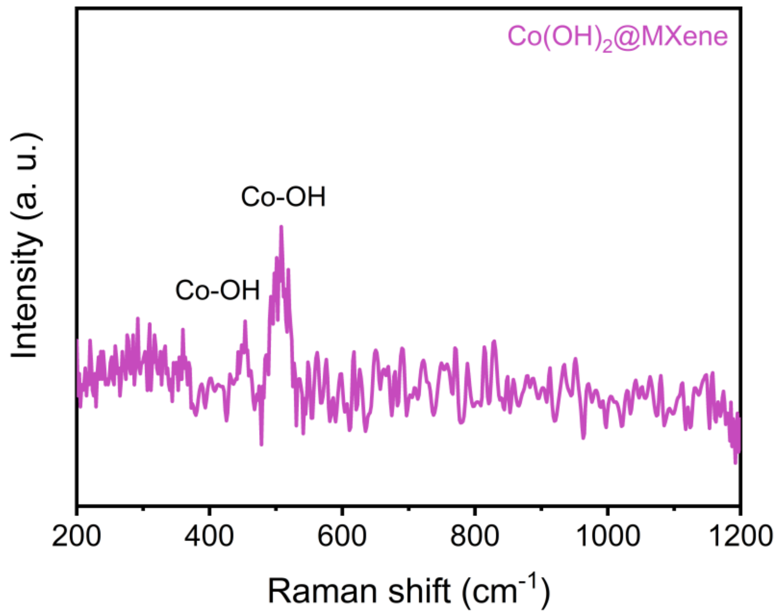

Supplementary Fig. 22. Raman analysis. Raman spectra of as-prepared $Co(OH)_2$@MXene.

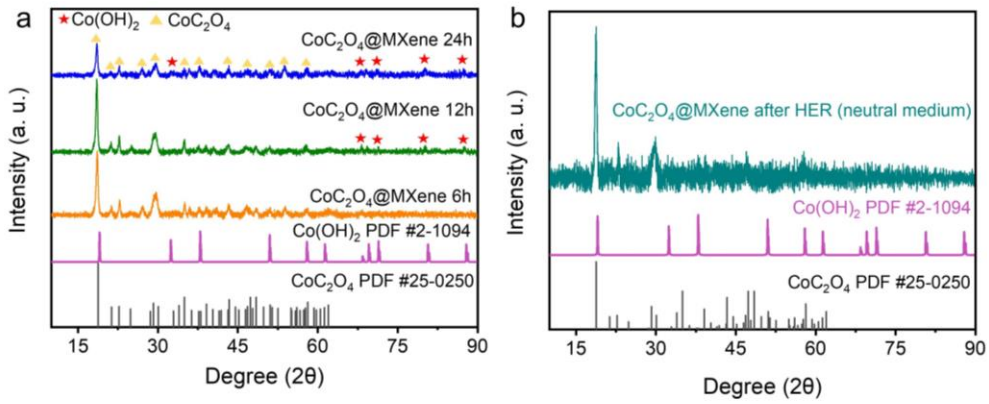

Supplementary Fig. 23. Phase characterization. a) XRD patterns of CoC_2O_4@MXene after soaking in 1M KOH for 6, 12, and 24 h, respectively. b) XRD patterns of CoC_2O_4@MXene after neutral HER.

The XRD of CoC_2O_4@MXene immersed in 1 KOH solution for a long time exhibits incomplete reconfiguration. The crystal phase of CoC_2O_4@MXene remains unchanged after neutral HER.

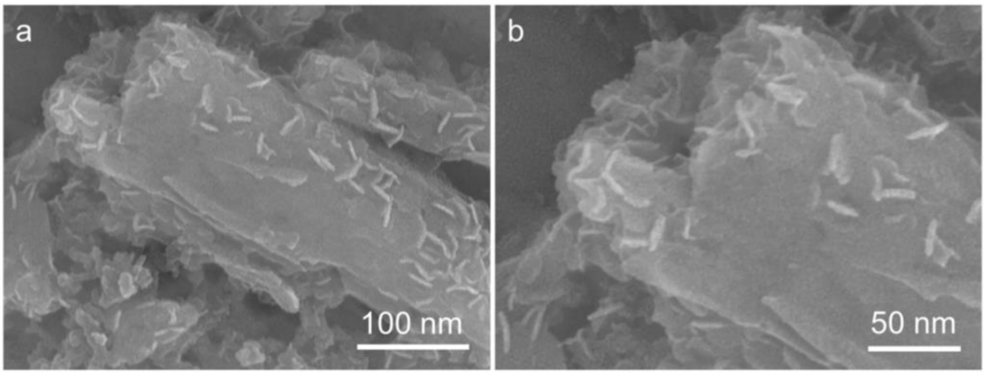

Supplementary Fig. 24. Morphology characterization. a) Low-magnified and b) high-magnified SEM images of CoC_2O_4@MXene soaked in 1 M KOH for 24 h, respectively.

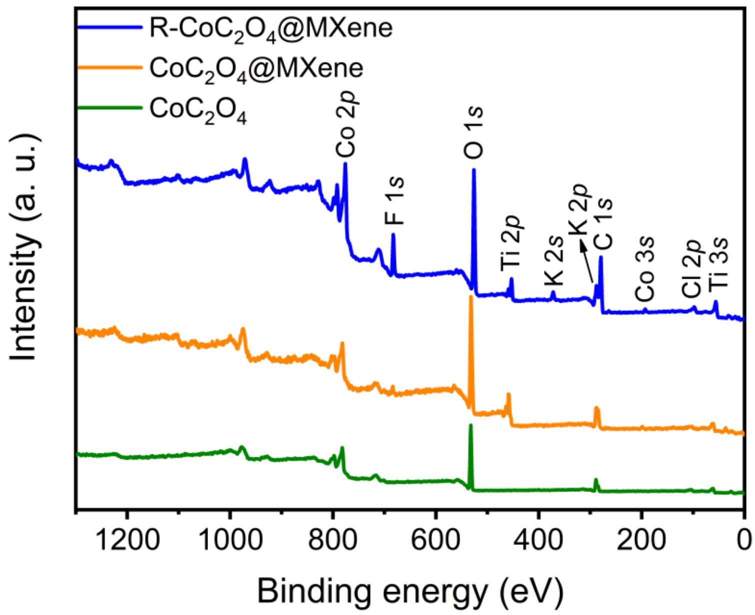

Supplementary Fig. 25. Surface elemental composition analysis. Comparison of full XPS survey spectra of CoC_2O_4, CoC_2O_4@MXene, and R-CoC_2O_4@MXene, respectively.

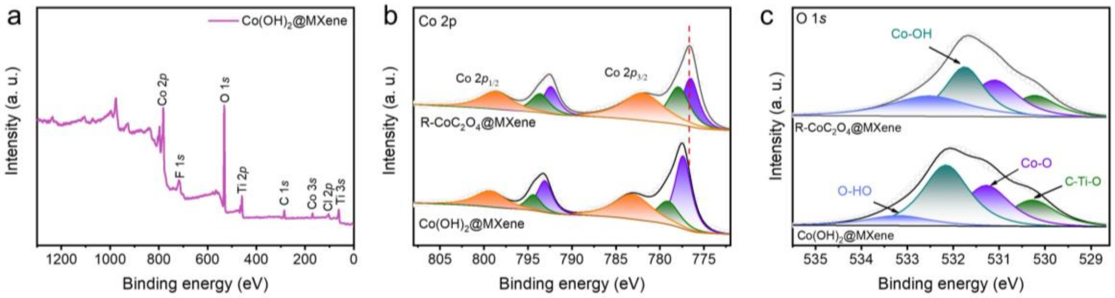

Supplementary Fig. 26. Surface elemental composition analysis. a) Full XPS survey spectrum of $Co(OH)_2$@MXene. XPS spectra of the b) Co 2*p* and c) O 1*s* regions for R-CoC_2O_4@MXene and $Co(OH)_2$@MXene, respectively.

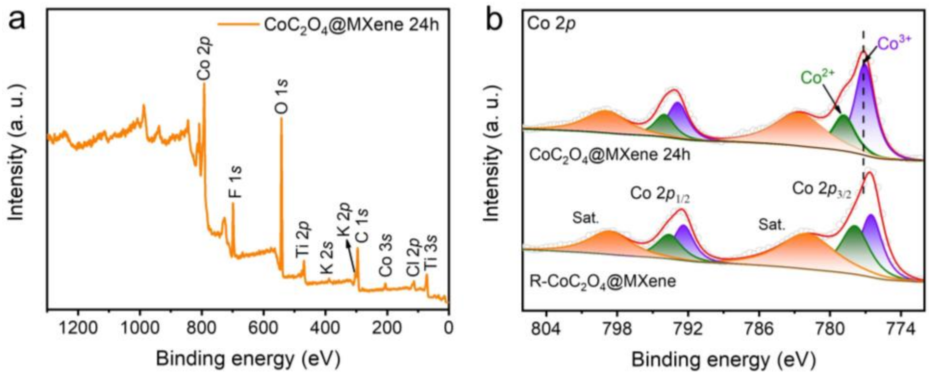

Supplementary Fig. 27. Surface elemental composition analysis. a) Full XPS survey spectrum of CoC_2O_4@MXene soaked in 1 M KOH for 24 h. b) XPS spectra of the Co 2*p* region of R-CoC_2O_4@MXene and CoC_2O_4@MXene soaked in 1 M KOH for 24 h, respectively.

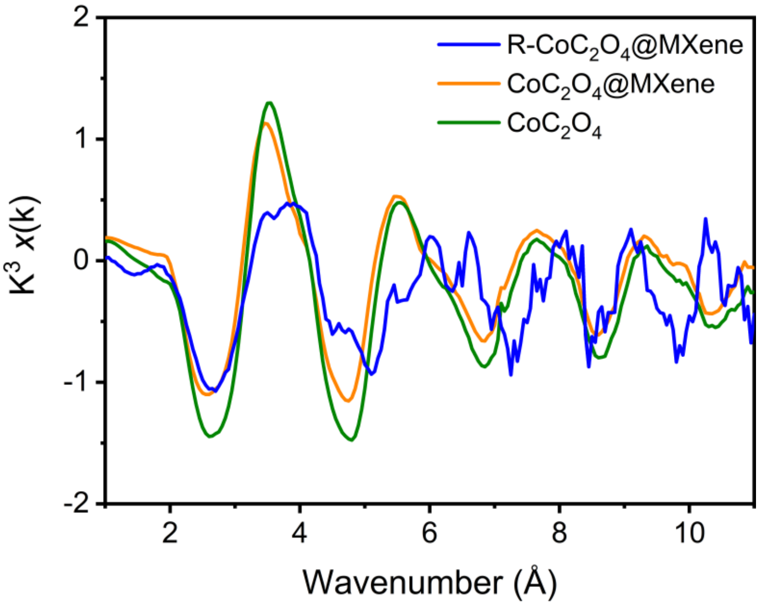

Supplementary Fig. 28. EXAFS curves of prepared catalysts. Co K-edge EXAFS oscillation function of CoC_2O_4, CoC_2O_4@MXene, and R-CoC_2O_4@MXene, respectively.

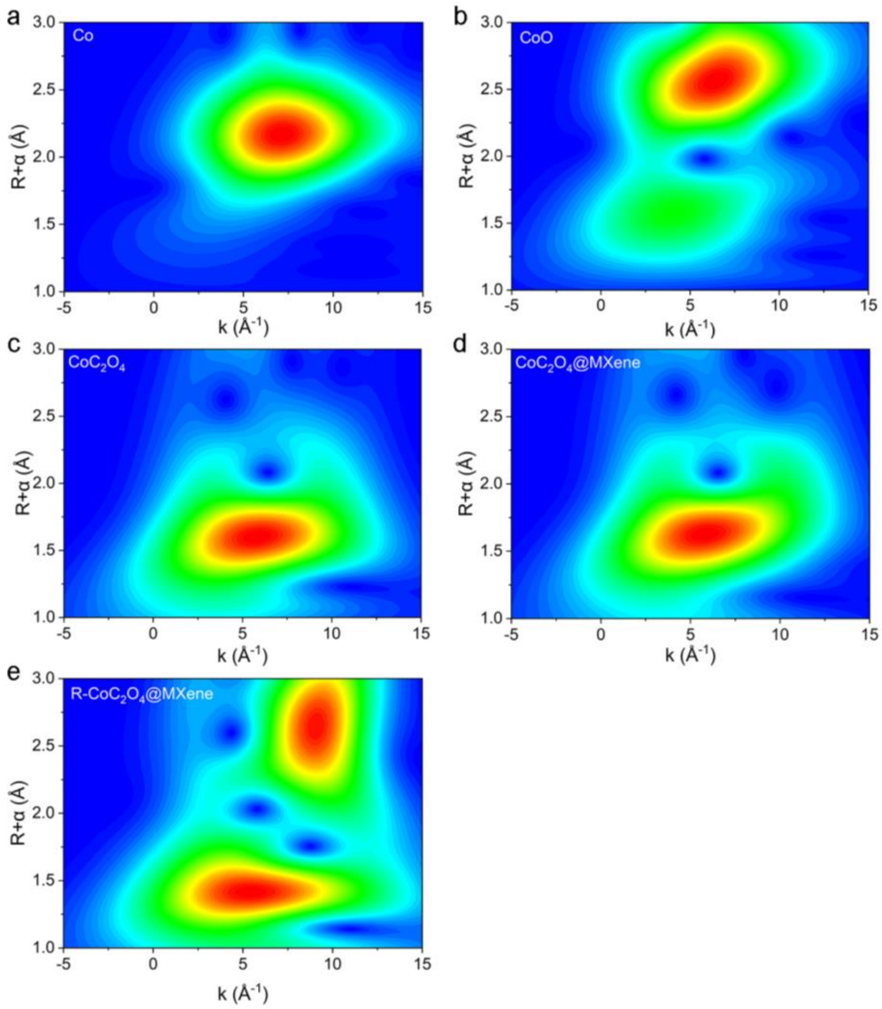

Supplementary Fig. 29. Wavelet transform of references. Wavelet transform for k^3-weighted EXAFS signal of a) Co foil, b) CoO, c) CoC_2O_4, d) CoC_2O_4@MXene, and e) R-CoC_2O_4@MXene, respectively.

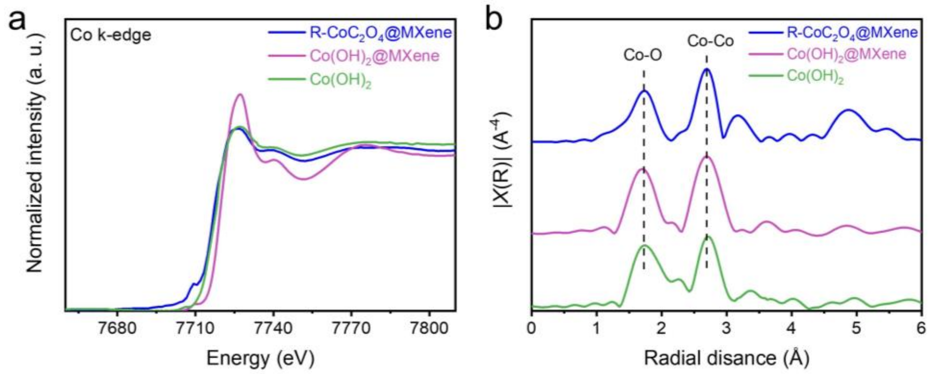

Supplementary Fig. 30. Electronic and fine structural characterizations. a) Co K-edge XANES and b) FT-EXAFS spectra of the R-CoC_2O_4@MXene, $Co(OH)_2$, and $Co(OH)_2$@MXene, respectively.

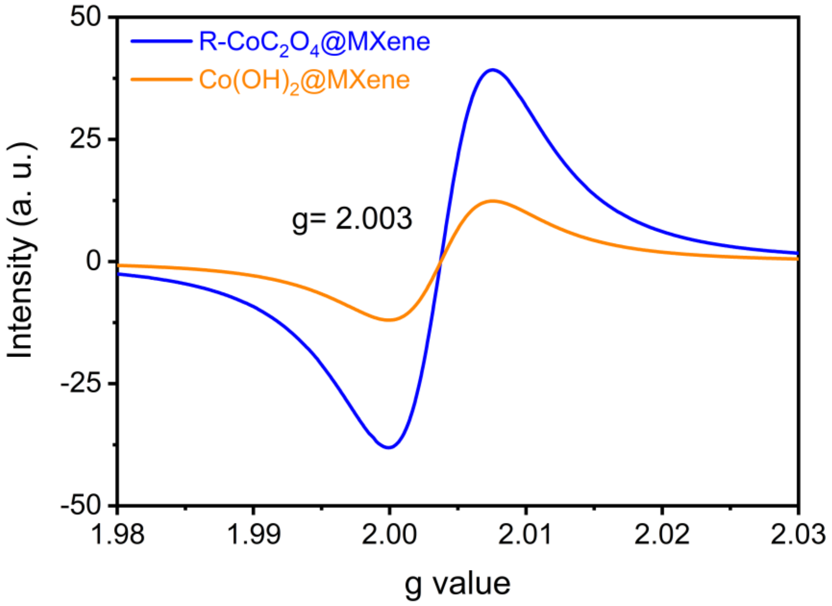

Supplementary Fig. 31. Oxygen vacancy characterization. EPR of R-CoC_2O_4@MXene and as-prepared $Co(OH)_2$@MXene, respectively.

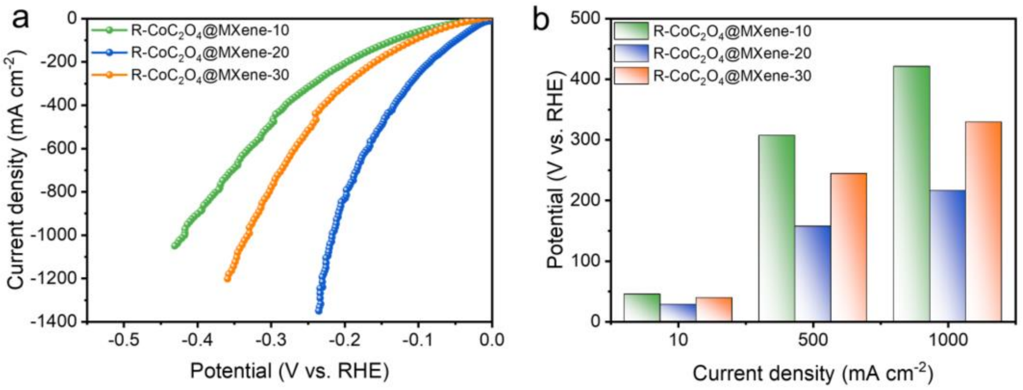

Supplementary Fig. 32. Study for the diffferent molar ratios of composites. a) HER LSV curves of R-CoC_2O_4@MXene with different ratios of 10, 20, and 30 mL MXene. b) Comparison of overpotentials required to achieve 10, 500, and 1000 mA cm^{-2} for R-CoC_2O_4@MXene-10, R-CoC_2O_4@MXene-20, and R-CoC_2O_4@MXene-30, respectively.

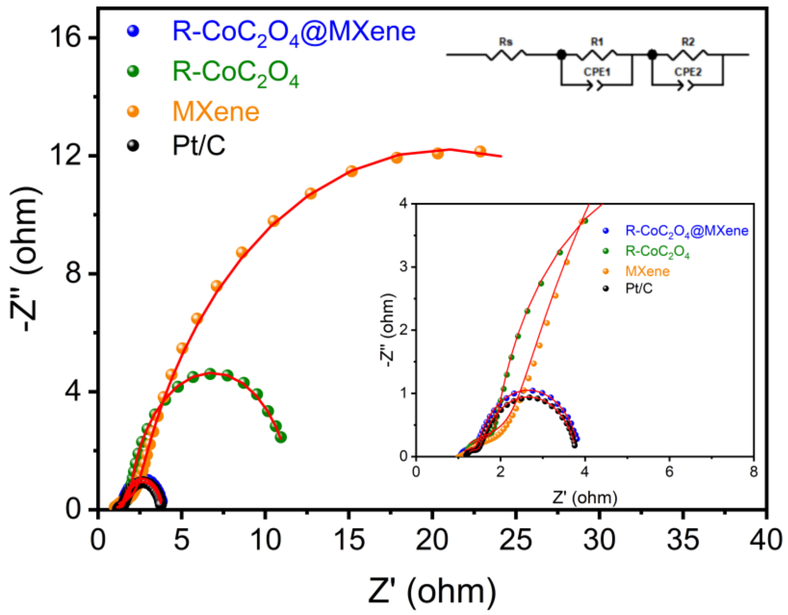

Supplementary Fig. 33. Electrochemical impedance spectra. EIS curves for HER of MXene, R-CoC_2O_4, R-CoC_2O_4@MXene, and Pt/C, respectively.

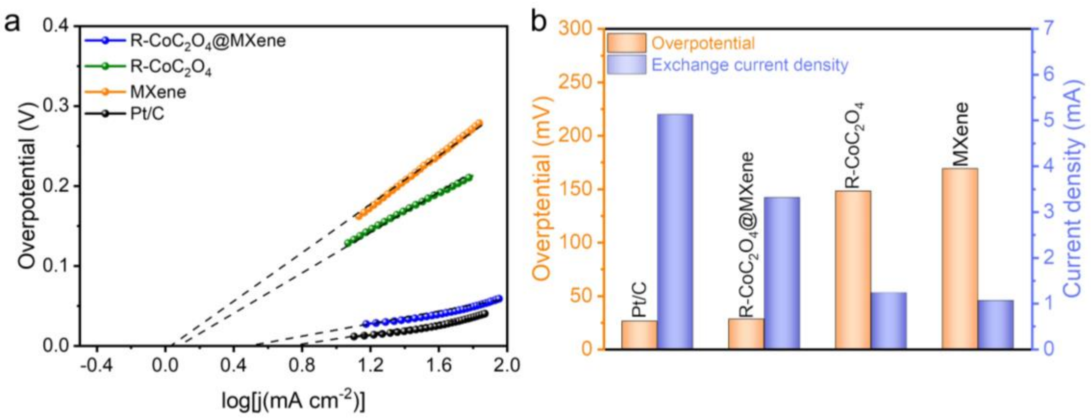

Supplementary Fig. 34. Exchange current density analysis. a) Exchange current densities in 1 M KOH calculated via extrapolation method. b) Overpotentials at 10 mA cm^{-2} (left) and exchange current densities (right) of MXene, R-CoC_2O_4, R-CoC_2O_4@MXene, and Pt/C in 1.0 M KOH, respectively.

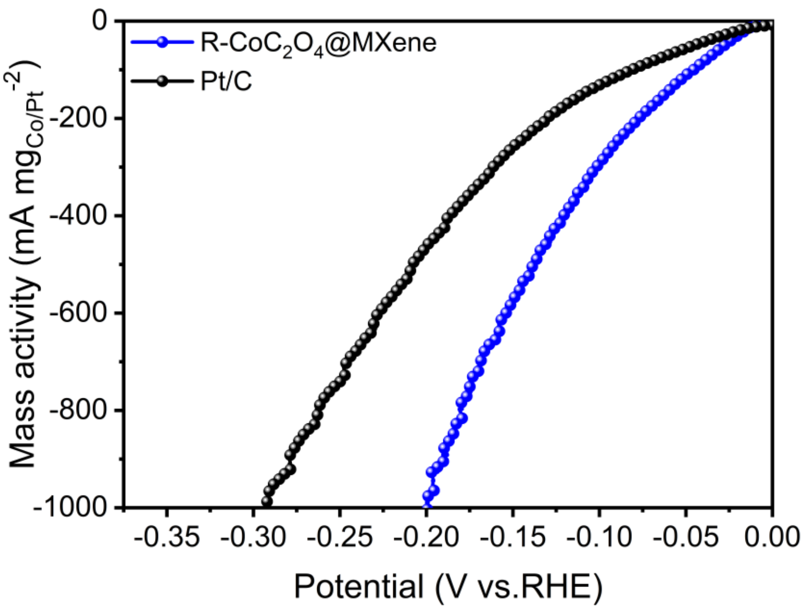

Supplementary Fig. 35. Normalized Mass activity. LSV curves of R-CoC_2O_4@MXene and Pt/C normalized by actual metal (Co or Pt) mass in 1 M KOH solution.

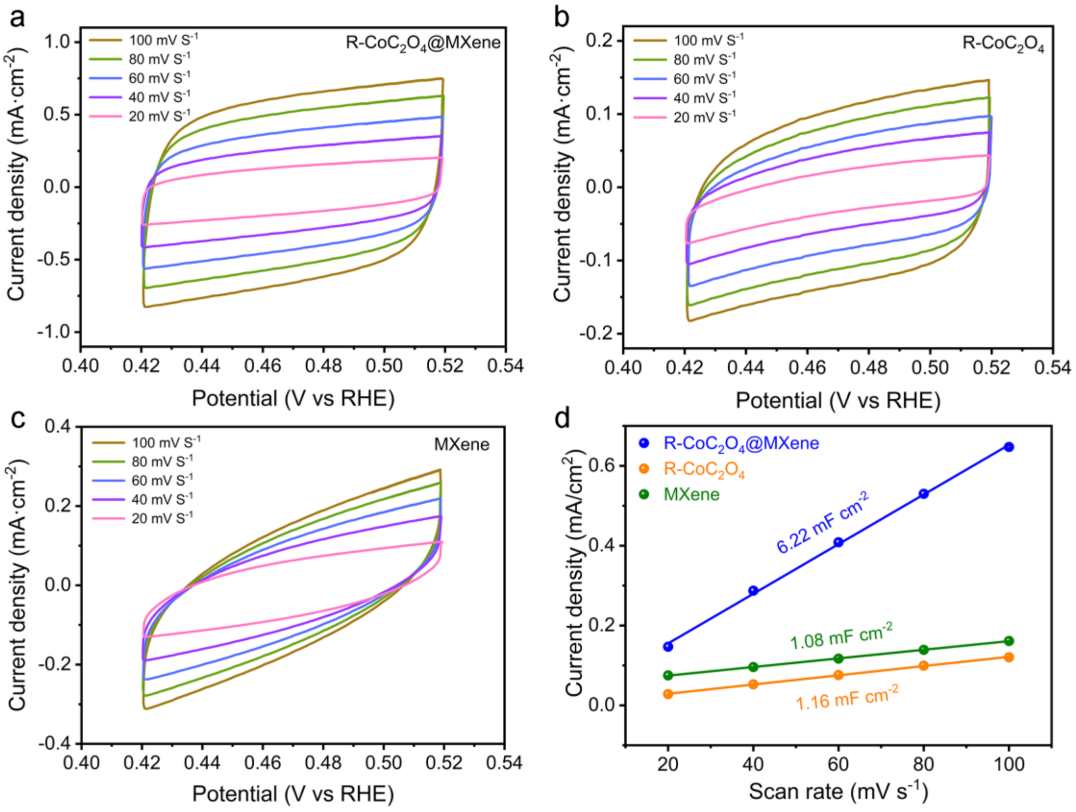

Supplementary Fig. 36. CV plots and C_{dl}. Cyclic voltammograms of a) R-CoC_2O_4@MXene, b) R-CoC_2O_4, and c) MXene at various scan rates (20, 40, 60, 80, and 100 mV s^{-1}) in the potential range of 0.42-0.52 V vs. RHE, which were used to estimate the double-layer capacitance (C_{dl}). d) Corresponding linear fitting of the current density versus scan rates.

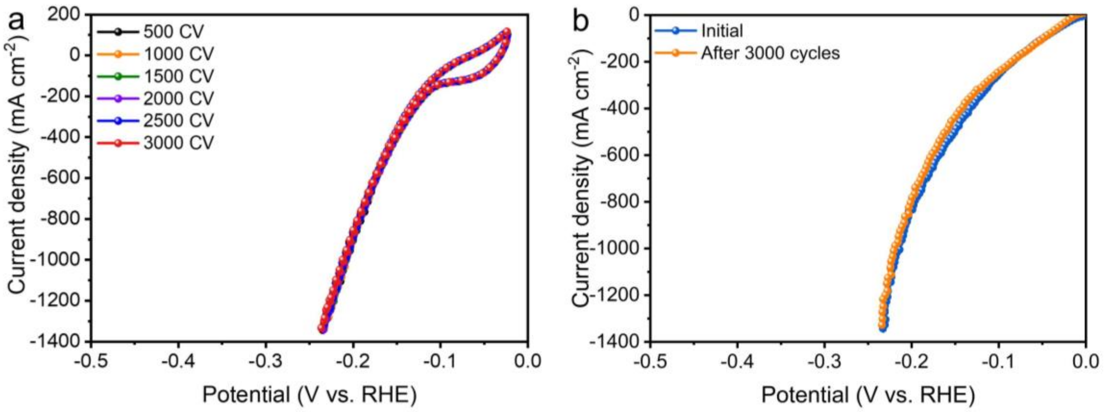

Supplementary Fig. 37. CV cycling test. a) 3000 cycles CV of R-CoC_2O_4@MXene. b) Polarization curves of R-CoC_2O_4@MXene at the initial stage and after 3000 cycles.

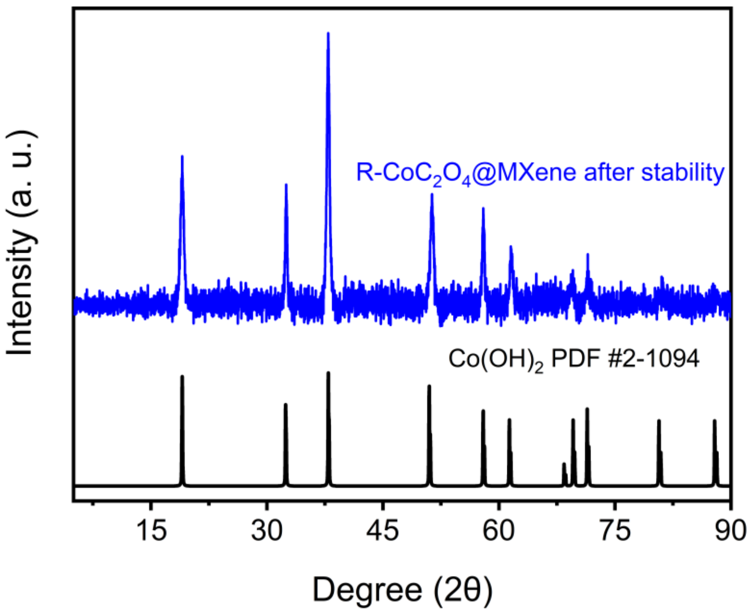

Supplementary Fig. 38. Phase characterization. XRD of R-CoC_2O_4@MXene after stability.

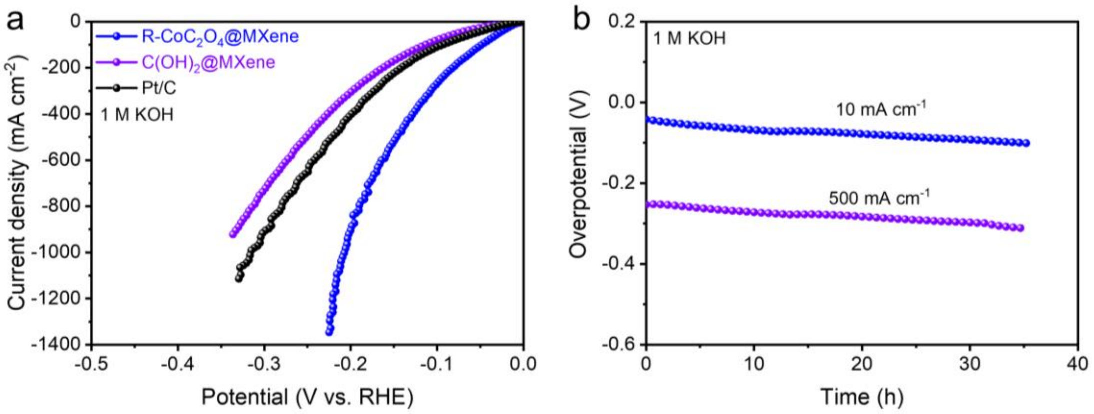

Supplementary Fig. 39. Electrochemical HER performance measurements. a) HER polarization curves of Pt/C, R-CoC_2O_4@MXene, and $Co(OH)_2$@MXene, respectively. b) Chronopotentiometry curves of $Co(OH)_2$@MXene at constant current densities of 10 and 500 mA cm^{-2}.

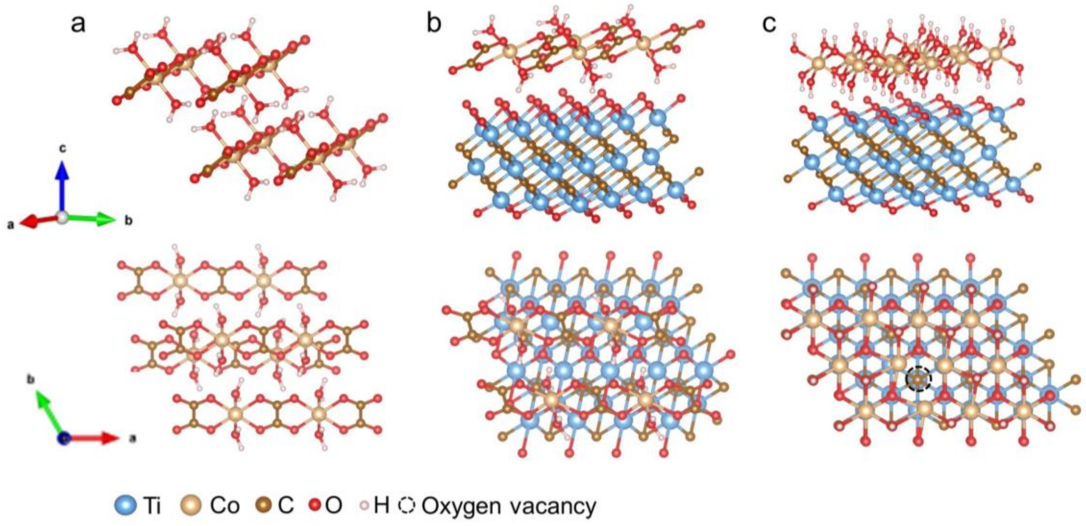

Supplementary Fig. 40. DFT calculated structural models. Top and side views of the structural models of a) CoC_2O_4, b) CoC_2O_4@MXene, and c) R-CoC_2O_4@MXene, respectively.

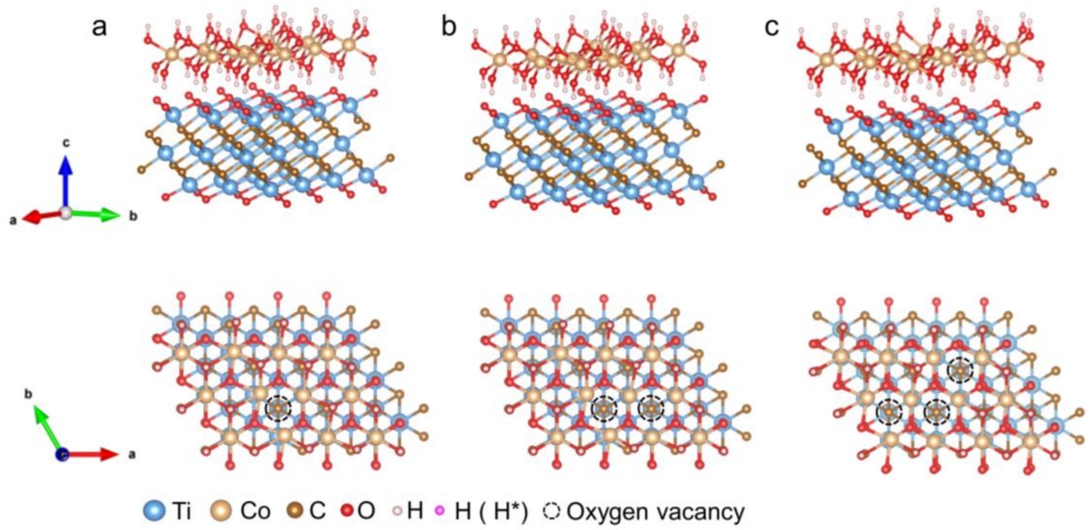

Supplementary Fig. 41. DFT calculated structural models. a-c) Top and side views of the structural models of R-CoC_2O_4@MXene with different oxygen defect contents.

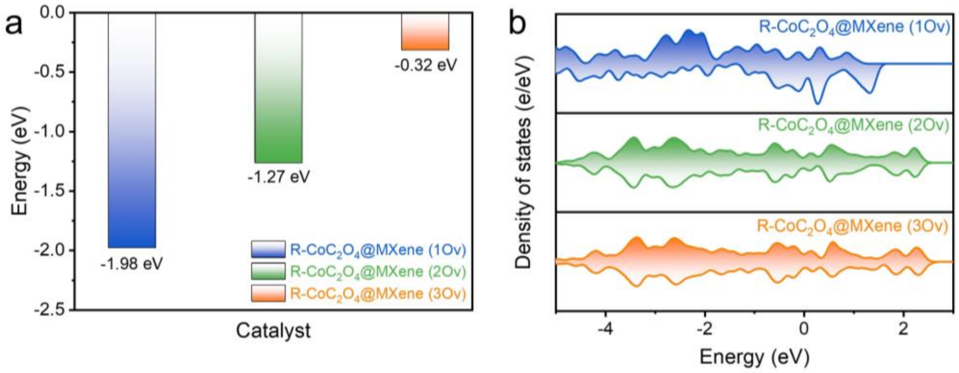

Supplementary Fig. 42. Formation energy and DOS analysis. a) Formation energy and b) DOS plots of the R-CoC_2O_4@MXene (1O_V), R-CoC_2O_4@MXene (2O_V), and R-CoC_2O_4@MXene (3O_V) structural models, respectively.

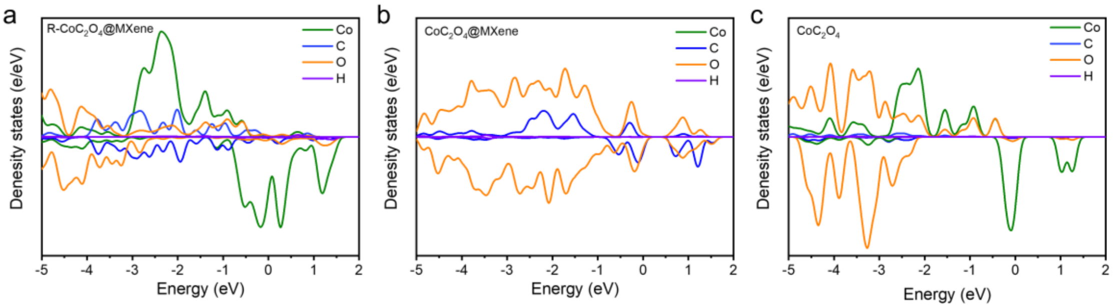

Supplementary Fig. 43. Density state analysis. Calculated density of states for a) CoC_2O_4, b) CoC_2O_4@MXene, and c) R-CoC_2O_4@MXene, respectively.

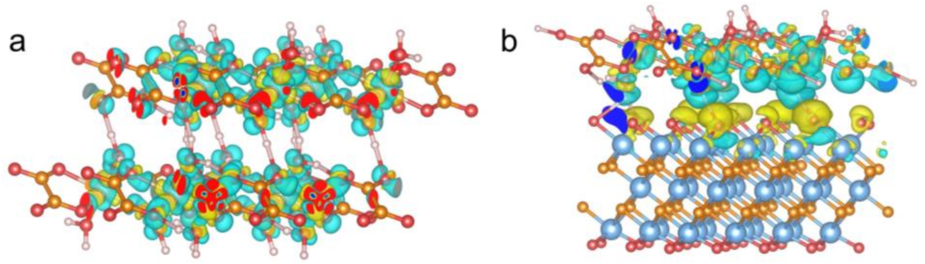

Supplementary Fig. 44. Charge density distribution. Differential charge density of a) CoC_2O_4 and b) CoC_2O_4@MXene respectively. The yellow and blue zones represent the charge accumulation or dispersion, respectively.

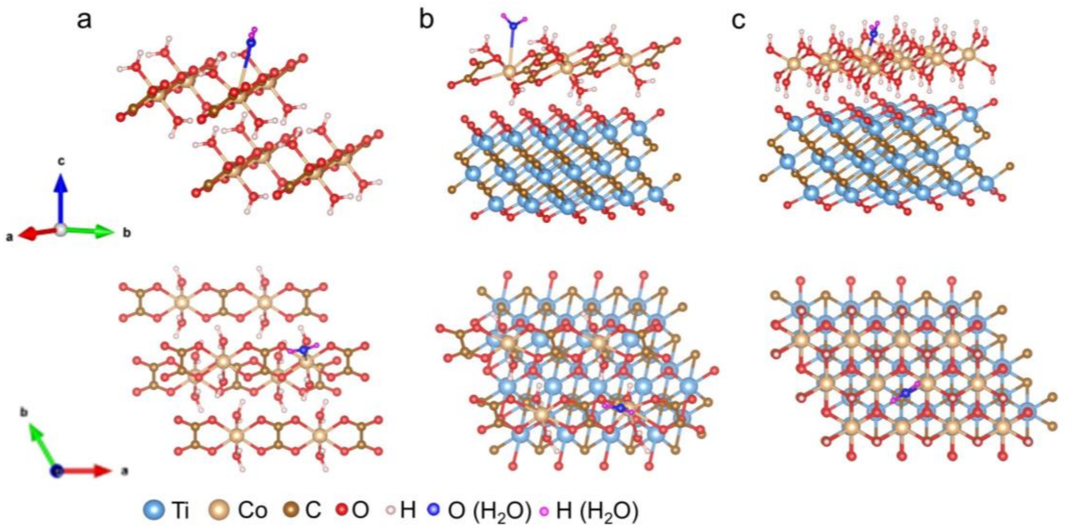

Supplementary Fig. 45. DFT calculated structural models. Top and side views of the simulated H_2O adsorption structures of a) CoC_2O_4, b) CoC_2O_4@MXene, and c) R-CoC_2O_4@MXene.

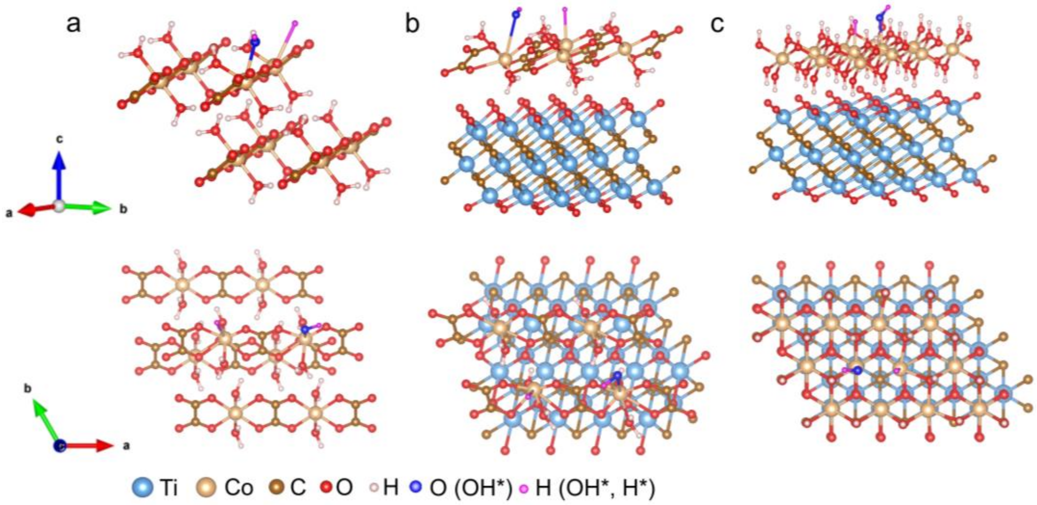

Supplementary Fig. 46. DFT calculated structural models. Top and side views of the simulated H_2O dissociation structures of a) CoC_2O_4, b) CoC_2O_4@MXene, and c) R-CoC_2O_4@MXene.

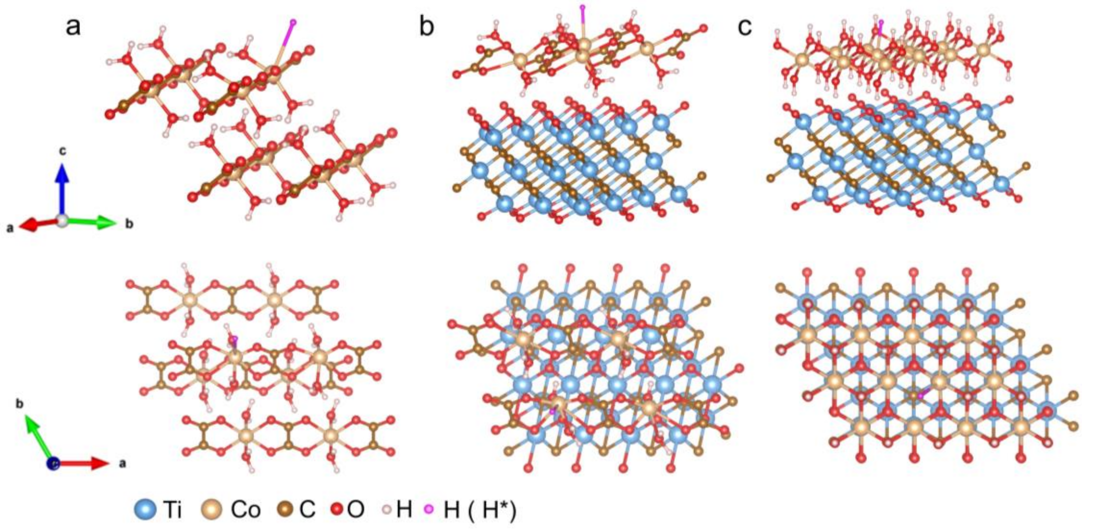

Supplementary Fig. 47. DFT calculated structural models. Top and side views of the simulated H adsorption structures of a) CoC_2O_4, b) CoC_2O_4@MXene, and c) R-CoC_2O_4@MXene.

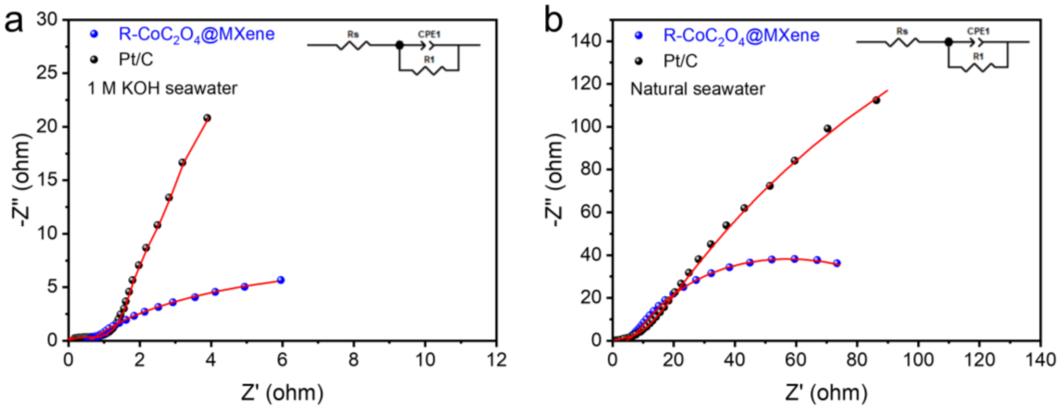

Supplementary Fig. 48. Electrochemical impedance spectra. Nyquist plots of R-CoC_2O_4@MXene electrodes in a) 1 M KOH seawater and b) neutral seawater, respectively.

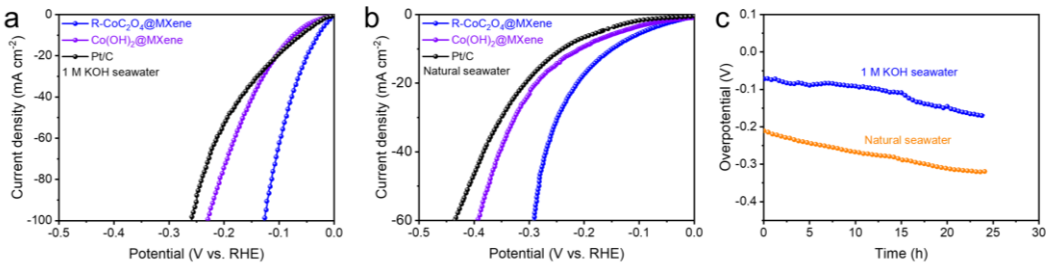

Supplementary Fig. 49. Electrochemical HER performance in seawater. a, b) The HER polarization curves of MXene, R-CoC_2O_4@MXene and $Co(OH)_2$@MXene catalysts in 1 M KOH seawater and natural seawater, respectively. c) Chronopotentiometry curve of $Co(OH)_2$@MXene in 1 M KOH seawater and natural seawater, respectively.

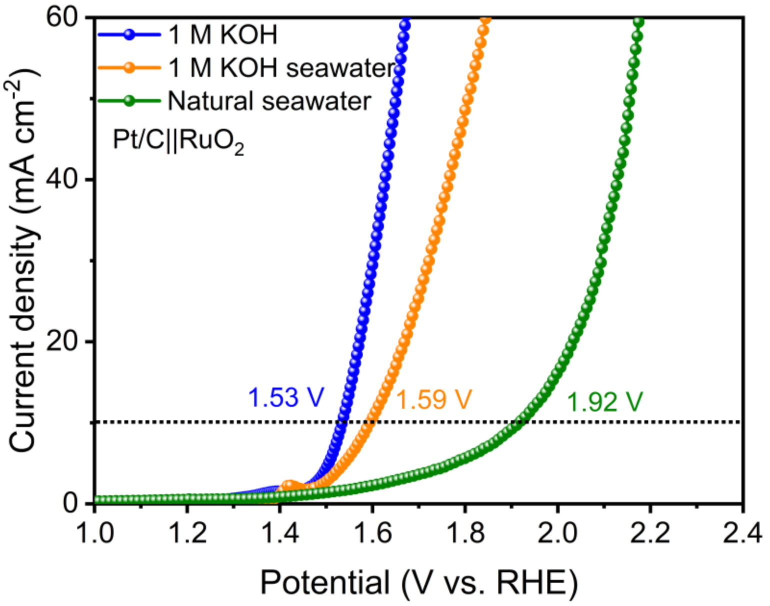

Supplementary Fig. 50. Water electrolysis performance measured in seawater. Polarization curve of the Pt/C||RuO_2 toward overall water splitting in different electrolytes.

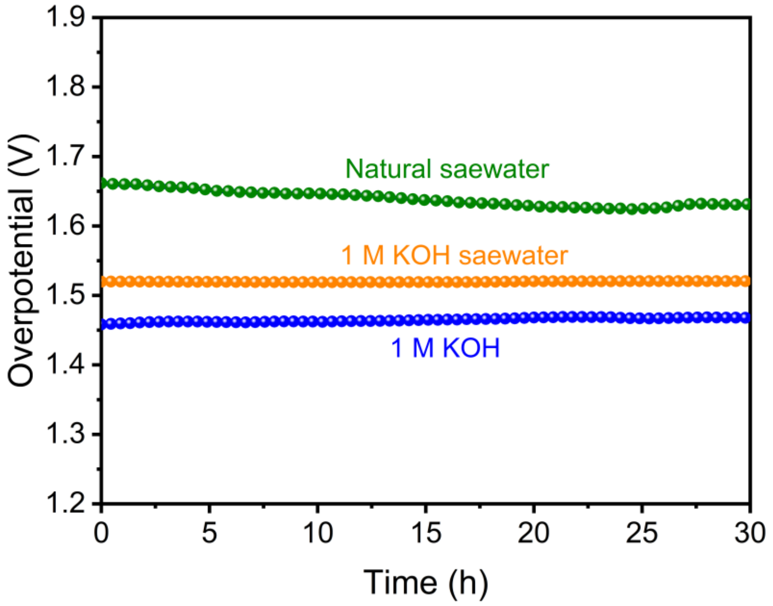

Supplementary Fig. 51. Electrolytic seawater stability. Long-term stability tests of R-CoC_2O_4@MXene conducted at a constant current density of 10 mA cm^{-2} in different electrolytes.

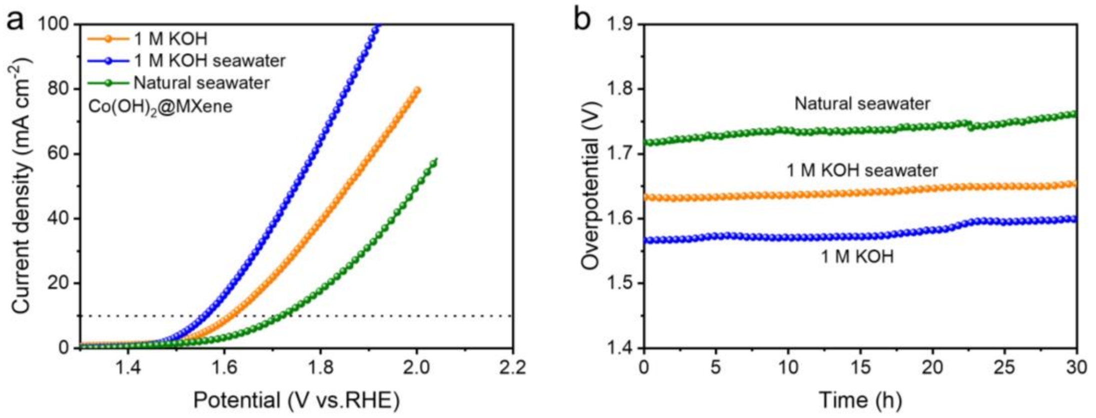

Supplementary Fig. 52. Water electrolysis performance measured in seawater. a) Polarization curve and b) long-term stability tests of the $Co(OH)_2$@MXene||$Co(OH)_2$@MXene toward overall water splitting in different electrolytes.

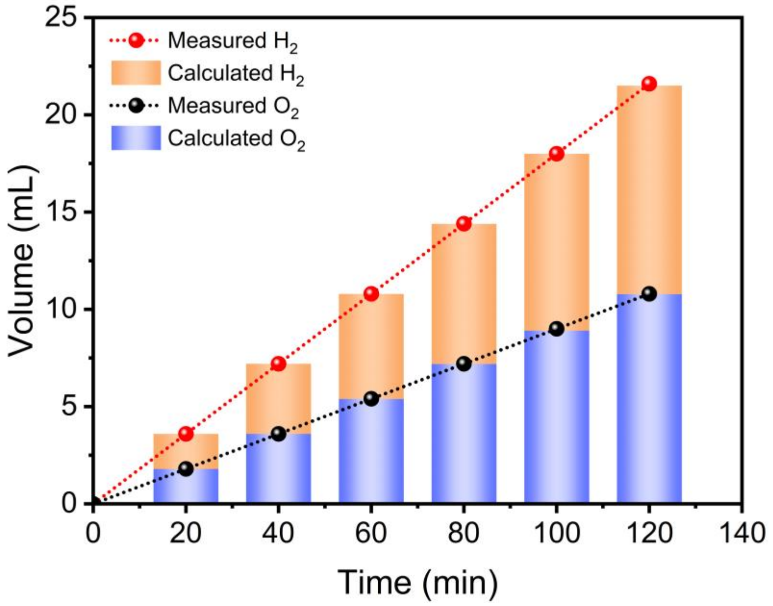

Supplementary Fig. 53. Faraday efficiency. Faraday efficiency of R-CoC_2O_4@MXene in 1 M KOH seawater.

Supplementary Methods

Turnover frequency (TOF) was calculated via the following formula according to previous reports.[1-3]

$$\text{TOF per site}=\frac{\text{\# Total Hydrogen Turn Over/cm}^2\text{ geometric area}}{\text{\# Surface Sites/cm}^2\text{geometric area}} \tag{1}$$

The total number of hydrogen turn overs was calculated from the current density using the following equation:[3]

$$\#H_2=\left(j\frac{\text{mA}}{\text{cm}^2}\right)\left(\frac{1\text{C s}^{-1}}{1000\text{ mA}}\right)\left(\frac{1\text{ mol s}^{-1}}{1000\text{ mA}}\right)\left(\frac{1\text{ mol H}_2}{2\text{ mol e}^-}\right)\left(\frac{6.022\times10^{23}\text{H}_2\text{ molecules}}{1\text{ mol H}_2}\right)$$

$$=3.12\times10^{15}\frac{\text{H}_2\text{/s}}{\text{cm}^2}\text{per}\frac{\text{mA}}{\text{cm}^2} \tag{2}$$

The total number of effective surface sites was calculated based on the following equation:

$$\frac{\text{\#Surface sites}}{\text{cm}^2\text{geometric area}}=\frac{\text{\#Surface sites (flat standzrd)}}{\text{cm}^2\text{geometric area}}\times\text{Roughness fator} \tag{3}$$

Here the roughness factor (RF) was calculated according to the following equation:[4]

$$\text{RF}=\frac{C_{dl}(\text{sample})}{C_{dl}(\text{flat standard})} \tag{4}$$

The double-layer capacitance (C_{dl}) of the different samples was calculated in Supplementary Fig. 36, and we considered a C_{dl} value of 60 μF cm^{-2} for a flat electrode,[4, 5] which is close to the range of expected values for a flat electrode (typically 20 ~ 60 μF cm^{-2}).[2] Thus, the RFs of the different catalysts are:

$$\text{RF(MXene)}:\frac{1.08\times10^3}{60}=18.0 \tag{5}$$

$$\text{RF(R-CoC}_2\text{O}_4\text{)}:\frac{1.16\times10^3}{60}=19.33 \tag{6}$$

$$\text{RF(R-CoC}_2\text{O}_4\text{@MXene)}:\frac{6.22\times10^3}{60}=103.66 \tag{7}$$

For flat standard samples, we assume that the active site density is the same for all three catalysts as an approximation. This approximation is reasonable given that all of the materials have a surface site density close to 2 × 10^{15} sites/cm^2. Thus, the numbers of surface-active sites for the different catalysts are:

$$\text{MXene}:18.0\times2\times10^{15}\text{surface}\frac{\text{sites}}{\text{cm}^2}=3.60\times10^{16}\text{ surface sites/cm}^2 \tag{8}$$

$$\text{R-CoC}_2\text{O}_4:19.33\times2\times10^{15}\text{surface}\frac{\text{sites}}{\text{cm}^2}=3.86\times10^{16}\text{ surface sites/cm}^2 \tag{9}$$

$$\text{R-CoC}_2\text{O}_4\text{@MXene}:103.66\times2\times10^{15}\text{surface}\frac{\text{sites}}{\text{cm}^2}=2.07\times10^{17}\text{ surface sites/cm}^2 \tag{10}$$

Therefore, the TOF per active site is calculated as follows:

$$\left(3.12\times10^{15}\frac{\text{H}_2\text{/s}}{\text{cm}^2}\Big/\frac{\text{mA}}{\text{cm}^2}\right)\left(|j|\frac{\text{mA}}{\text{cm}^2}\right)\left(\frac{1\text{ cm}^2}{3.60\times10^{16}\text{surface sites}}\right)=0.0866|j|\frac{\text{H}_2\text{/s}}{\text{surface site}} \tag{11}$$

$$\left(3.12\times10^{15}\frac{H_2/s}{cm^2}\Big/\frac{mA}{cm^2}\right)\left(|j|\frac{mA}{cm^2}\right)\left(\frac{1\ cm^2}{3.86\times10^{16}surface\ sites}\right)=0.0808|j|\frac{H_2/s}{surface\ site} \tag{12}$$

$$\left(3.12\times10^{15}\frac{H_2/s}{cm^2}\Big/\frac{mA}{cm^2}\right)\left(|j|\frac{mA}{cm^2}\right)\left(\frac{1\ cm^2}{2.07\times10^{17}surface\ sites}\right)=0.0150|j|\frac{H_2/s}{surface\ site} \tag{13}$$

for MXene, CoC_2O_4, and CoC_2O_4@MXene, respectively.

At an overpotential of 200 mV, the current densities are 28, 41, and 766 mA cm^{-2} for the MXene, R-CoC_2O_4, and R-CoC_2O_4@MXene catalysts, respectively. Thus, the TOFs of the different catalysts at an overpotential of 200 mV are:

$$\text{MXene: TOF}=0.0866|j|\frac{H_2/s}{surface\ site}=0.0866\times28\ S^{-1}=2.424\ S^{-1} \tag{14}$$

$$\text{R–}CoC_2O_4\text{: TOF}=0.0808|j|\frac{H_2/s}{surface\ site}=0.0808\times41\ S^{-1}=3.312\ S^{-1} \tag{15}$$

$$\text{R–}CoC_2O_4\text{@MXene: TOF}=0.0150|j|\frac{H_2/s}{surface\ site}=0.0150\times766\ S^{-1}=11.490\ S^{-1} \tag{16}$$

Supplementary Tables

Supplementary Table 1. Summary of reconfiguration product, reconfiguration degree, reconfiguration time, and HER performance of different pre-catalysts in 1 M KOH.

Pre-catalyst	Reconfiguration product	Reconfiguration degree	Reconfiguration time	Overpotential (mV) 10 mA cm^{-2}	Stability (h)	References
**CoC_2O_4@MXene**	**$Co(OH)_2$@MXene**	**Complete reconfiguration**	**5 cycles LSV (8.3 min)**	**28**	**100@10 mA cm^{-2}**	**This Work**
Ni_4Mo	Ni_4Mo@$Mo_2O_7^{2-}$	Surface reconfiguration	4 h	30	50@58 mA cm^{-2}	Angew. Chem. Int. Ed. 2021, 60, 7051-7055.
$NiSe_2$	$NiSe/NiSe_2$	Deep reconfiguration	20 cycles LSV	50	25@160 mV vs RHE	ACS Energy Lett. 2020, 5, 2483-2491.
$TiO_2|Co_2P_4O_{12}$	$CoO_x(OH)_y/TiO_2|Co_2P_4O_{12}$	Surface reconfiguration	35 cycles LSV	50	40@90 mV vs RHE	J. Mater. Chem. A. 2019, 7, 12457-12467.
CoF_2	$Co(OH)_2$	Complete reconfiguration	60 cycles LSV	54	110@10 mA cm^{-2}	Adv. Sci. 2022, 9, 2103567.
$Co_{3-x}Ni_xO_4$	$Co_yNi_{1-y}O/Co_{3-x}Ni_xO_4$	Surface reconfiguration	20 h	57	300@20 mA cm^{-2}	ACS Catal. 2021, 11, 8174-8182.
Mo_2C-Mo(VI)O_x/CC	Mo_2C-Mo(IV/VI)O_x/CC	Surface reconfiguration	100 cycles LSV	60	25@200 mV vs RHE	Angew. Chem. Int. Ed. 2020, 59, 3544-3548.
TiO_2@CoCH	$Co(OH)_2/TiO_2$@CoCH	Surface reconfiguration	25 cycles LSV	66	40@50 mV vs RHE	Nano Energy. 2021, 82, 105732.
NF-NiS_2	Ni/NF-NiS_2	Surface reconfiguration	100 cycles LSV	67	40@20 mA cm^{-2}	Nano Energy. 2017, 41, 148-153.
Ni-BDT	NiNSs/ Ni-BDT	Surface reconfiguration	1000 cycles LSV	80	30@20 mA cm^{-2}	Chem. 2017, 3, 122-133.
CoFeO@BP	CoP/CoFeO@BP	Surface reconfiguration	20 cycles LSV	88	22.5@90 mV vs RHE	Angew. Chem. Int. Ed. 2020, 59, 21106-21113.
$CoSe_{1.26}P_{1.42}$	Co/$CoSe_{1.26}P_{1.42}$	Deep reconfiguration	N.A.	92	15@92 mV vs RHE	ACS Energy Lett. 2019, 4, 987-994.
CoP	$Co(OH)_x$/CoP	Surface reconfiguration	10 h	100	25@92 mV vs RHE	Chem. Sci. 2019, 10, 2019-2024.
$Co_{0.5}W_{0.5}S_x$	$CoO/Co(OH)_2/Co_{0.5}W_{0.5}S_x$	Surface reconfiguration	10 h	110	10	J. Mater. Chem. A. 2021, 9, 11359-11369.
CoNP@C	$Co(OH)_2$/CoNP@C	Surface reconfiguration	3 h	191	20@20 mA cm^{-2}	Nano Energy. 2018, 49, 14-22.

Supplementary Table 2. Co K-edge EXAFS fitting of R-CoC_2O_4@MXene, CoC_2O_4@MXene, CoC_2O_4, CoO, and Co foil.

R is the bond distance; CN is coordination number; σ^2 is the Debye-Waller factor; ΔE_0 is adjustable "muffin-tin zero"; R factor is goodness of fit.

Sample	Path	CN	R (Å)	σ^2 ($Å^{-2}$)	ΔE_0 (eV)	R factor
R-CoC_2O_4@MXene	Co-O	5.5	2.071	0.0173	-0.282	0.0012
	Co-Co	6	3.097	0.0094	-0.282	
CoC_2O_4@MXene	Co-O	5.9	2.075	0.0175	-0.322	0.0013
	Co-Co	6	2.658	0.0093	-0.322	
CoC_2O_4	Co-O	5.9	2.072	0.0172	-0.412	0.0011
	Co-Co	6	2.652	0.0096	-0.412	
CoO	Co-O	6	2.098	0.0110	-0.531	0.0008
	Co-Co	12	3.125	0.0091	-0.531	
Co foil	Co-Co	12	2.585	0.0093	-0.432	0.0006

Supplementary Table 3. The fitting impedance parameters of MXene, R-CoC_2O_4, R-CoC_2O_4@MXene, and Pt/C in 1 M KOH, respectively.

Samples	Rs	R1	CPE1		R2	CPE2	
			CPE1-T	CPE1-P		CPE2-T	CPE2-P
R-CoC_2O_4@MXene	1.029	0.469	0.047	0.547	2.370	0.109	0.915
R-CoC_2O_4	1.130	0.846	0.011	0.621	9.732	0.014	0.967
MXene	1.832	1.620	0.021	0.497	36.832	0.022	0.870
Pt/C	1.172	0.358	0.043	0.592	2.279	0.156	0.877

Supplementary Table 4. The electrochemical active surface area of MXene, R-CoC_2O_4, and R-CoC_2O_4@MXene.

Sample	Slope value/mF cm^{-2}	ECSA/cm^2
MXene	1.08	27.00
R-CoC_2O_4	1.16	29.00
R-CoC_2O_4@MXene	6.22	155.50

Supplementary Table 5. Comparison of HER activity measured for R-CoC_2O_4@MXene with other representatives reported HER catalysts using 1.0 M KOH as electrolyte.

Sample	Overpotential (mV) 10 mA cm^{-2}	Overpotential (mV) 500 mA cm^{-2}	Overpotential (mV) 1000 mA cm^{-2}	Stability (h)	References
**R-CoC_2O_4@MXene**	**28**	**157**	**216**	100@10, 500, 1000 mA cm^{-2}	**This work**
2H $Nb_{1.35}S_2$	46	247	370	120@1000 mA cm^{-2}	Nat Mater. 2019, 18, 1309-1314.
W_1Mo_1-NG DAC	67	N.A.	N.A.	27@10 mA cm^{-2}	Sci. Adv. 2020, 6, eaba6586.
NFP/C-3	95	N.A.	N.A.	12@10 mA cm^{-2}	Sci. Adv. 2019, 5, eaav6009.
$Fe(OH)_x$@Cu-MOF NBs	112	N.A.	N.A.	30@112 mV vs RHE	Sci. Adv. 2021, 7, eabg2580.
$NiMoO_x$/NiMoS	38	186	240	25@500 mA cm^{-2}	Nat. Commun. 2020, 11, 5462.
MoS_2/Mo_2C	45	191	220	24@200 mA cm^{-2}	Nat. Commun. 2019, 10, 269.
FeP/Ni_2P	85	230	265	24@100 mA cm^{-2}	Nat. Commun. 2018, 9, 2551.
CF/VMFP	45	230	N.A.	30@250 mA cm^{-2}	Nat. Commun. 2021, 12, 1380.
MoC-Mo_2C-790	98.2	292	N.A.	50@500 mA cm^{-2}	Nat. Commun. 2021, 12, 6776.
HC-MoS_2/Mo_2C	280	320	412	24@400 mV vs RHE	Nat Commun. 2020, 11, 3724.
PW-Co_3N NWA/NF	41	N.A.	N.A.	25@92 mV vs RHE	Nat. Commun. 2020, 11, 1853.
Ni-Fe NP	46	N.A.	N.A.	24@10 mA cm^{-2}	Nat. Commun. 2019, 10, 5599.
NiCo-SAD-NC	61	N.A.	N.A.	15@100 mA cm^{-2}	Nat Commun. 2021, 12, 6766.
3CoMo-Vs	75	N.A.	N.A.	10@10 mA cm^{-2}	Nat Commun. 2020, 11, 2253.
$1T_{0.81}$-MoS_2@NiS_2	95	N.A.	N.A.	16@10 mA cm^{-2}	Nat Commun. 2021, 12, 5260.
Co-doped CeO_2	75	240	N.A.	14@150 and 200 mV vs RHE	J. Am. Chem. Soc. 2020, 142, 6461-6466.
Ni_2P/NF	85	245	300	10@2500 mA cm^{-2}	J. Am. Chem. Soc. 2019, 141, 7537-7543.
Co-NC-AF	85	272	343	32@1000 mA cm^{-2}	Adv.Mater. 2021, 33, 2103533.
P-Fe_3O_4/IF	39	212	242	20@100, 300, and 500 mA cm^{-2}	Adv. Mater. 2019, 31, 1905107.
$Cr_{0.4}Mo_{0.6}B_2$	180	390	N.A.	25@200 mV vs RHE	Adv. Mater. 2020, 32, 2000855.
CuCo-CAT/CC	52	N.A.	N.A.	10@10 mA cm^{-2}	Adv. Mater. 2021, 33, 2106781.

CoNi-inf	72	N.A.	N.A.	14@10 mA cm^{-2}	Adv. Mater. 2020, 32, 2002857.
$Ni/Ni(OH)_2$	77	N.A.	N.A.	10@10 mA cm^{-2}	Adv. Mater. 2020, 32, 1906915.
Ni@N-HCGHF	95	N.A.	N.A.	10@10 mA cm^{-2}	Adv. Mater. 2020, 32, 2003313.
MoO_2-FeP@C	103	N.A.	N.A.	24@10 mA cm^{-2}	Adv. Mater. 2020, 32, 2000455.
Ni_4Mo	34	N.A.	N.A.	50@58 mA cm^{-2}	Angew. Chem. Int. Ed. 2021, 60, 7051-7055.
Ni_3N-Co_3N PNAs/NF	43	N.A.	N.A.	40@25 mA cm^{-2}	Angew. Chem. Int. Ed. 2021, 60, 5984-5993.
S-$NiFe_2O_4$	61	N.A.	N.A.	60@200 mA cm^{-2}	Angew. Chem. Int. Ed. 2021, 60, 14117-14123.
Co-MoS_2@CoS_2	76	N.A.	N.A.		Angew. Chem. Int. Ed. 2022, 61, e2021148.
CoFeO@BP	88	N.A.	N.A.	22.5@10 mA cm^{-2}	Angew. Chem. Int. Ed. 2020, 59, 21106-21113.
P-MoP/Mo_2N	89	N.A.	N.A.	48@20 mA cm^{-2}	Angew. Chem. Int. Ed. 2021, 60, 6673-6681.
CoP_x@CNS	91	N.A.	N.A.	60@16 mA cm^{-2}	Angew. Chem. Int. Ed. 2020, 59, 21360-21366.
N-c-$CoSe_2$	98	N.A.	N.A.	24@130 mV vs RHE	Angew. Chem. Int. Ed. 2021, 60, 21575-21582.
S-$Co_{0.85}Se$-1	108	N.A.	N.A.	48@10 mA cm^{-2}	Angew. Chem. Int. Ed. 2021, 60, 12360-12365.
2D meso-Mo_2C/Mo_2N	110	N.A.	N.A.	20@10 mA cm^{-2}	Angew. Chem. Int. Ed. 2022, 134, e202112298.
NiP_2-650	134	N.A.	N.A.	14@50 mA cm^{-2}	Angew. Chem. Int. Ed. 2021, 60, 259-267.
Co-MoS_2	137	N.A.	N.A.	N.A.	Angew. Chem. Int. Ed. 2021, 60, 7251-7258.
$Co_{0.9}Ni_{0.1}Se$	185.7	N.A.	N.A.	N.A.	Angew. Chem. Int. Ed. 2020, 59, 22743-22748.
Co-Mo_5N_6	19	206	280	14@50 mA cm^{-2}	Adv. Energy Mater. 2020, 10, 2002176.
C-$Ni_{1-x}O$/3DPNi	32	220	245	10@10, 50, 1000 mA cm^{-2}	Adv. Energy Mater. 2020, 10, 2002955.
Ni/FeOOH	38	287	N.A.	18@100 mA cm^{-2}	Adv. Energy Mater. 2020, 10, 1904020.
NFN-MOF/NF	87	293	N.A.	30@250, 500 mA cm^{-2}	Adv. Energy Mater. 2018, 8, 1801065.
E-Co SAs	59	280	N.A.	500@10 mA cm^{-2}	Adv. Funct. Mater. 2021, 31, 2100547.
Ni-Mo-B HF	23	329	N.A.	300@450 mA cm^{-2}	Adv. Funct. Mater. 2021, 32, 2107308.
PS-Cu	121	750	980	30@100 mA cm^{-2}	Adv. Funct. Mater. 2022, 32, 2112367.

NiMnOP/NF	81	195	N.A.	35@500 mA cm^{-2}	Nano Energy. 2020, 69, 104432.
NiFe-LDH/MXene/NF	132	205	N.A.	280@10 mA cm^{-2}	Nano Energy. 2019, 63, 103880.
MFN-MOFs(2:1)/NF	79	234	N.A.	100@500 mA cm^{-2}	Nano Energy. 2019, 57, 1-13.
Ni_2P-CuP_2	51	360	460	75@100, 200, 500 mA cm^{-2}	ACS Nano. 2021, 15, 5586-5599.
$Ni_3Sn_2S_2$@Ni_3S_2	50	206	N.A.	20@200 mA cm^{-2}	Appl. Catal. B: Environ. 2020, 267, 118675.
NiFeMo IOS@NF	33	249	N.A.	50@500 mA cm^{-2}	Appl. Catal. B: Environ. 2020, 267, 118376.
A-NiCo LDH/NF	43	286	381	72@291, 381 mV vs RHE	Appl. Catal. B: Environ. 2020, 261, 118240.
IrFe/NC	37	510	850	12@100 mA cm^{-2}	Appl. Catal. B: Environ. 2019, 258, 117965.
NF@NiFeLDH	166	356	N.A.	120@300 mA cm^{-2}	Small. 2021, 18, 2104354.

Supplementary Table 6. The fitting impedance parameters of MXene, R-CoC_2O_4, R-CoC_2O_4@MXene, and Pt/C in 1 M KOH seawater and neutral seawater, respectively.

Samples	Rs	R1	CPE1	
			CPE1-T	CPE1-P
R-CoC_2O_4@MXene (1 M KOH seawater)	1.034	17.764	0.146	0.784
Pt/C (1 M KOH seawater)	1.125	35.236	0.156	0.569
R-CoC_2O_4@MXene (Natural seawater)	0.958	68.412	0.126	0.657
Pt/C (Natural seawater)	1.021	102.351	0.115	0.548

Supplementary Table 7. Comparison of HER activity measured for R-CoC_2O_4@MXene with other representatives reported HER catalysts using alkaline seawater as electrolyte.

Sample	Overpotential (mV) 10 mA cm^{-2}	Stability (h)	Electrolyte	References
**R-CoC_2O_4@MXene**	**32**	**100**	**1 M KOH seawater**	**This work**
NiMoN	40	48	1 M KOH seawater	Nat. Commun. 2019, 10, 5106.
Ni-NiO-Cr_2O_3	41	22	Simulated alkaline seawater	Proc. Natl. Acad. Sci. U.S.A. 2019, 116, 6624-6629.
C-Co_2P	42	60	Simulated alkaline seawater	Adv. Funct. Mater. 2021, 31, 2107333.
NiFeP-NS	45	10	Simulated alkaline seawater	Appl. Catal. B: Environ. 2022, 302, 120862.
MIL-(IrNiFe)@NF	47	24	1 M KOH seawater	J. Mater. Chem. A. 2021, 9, 27424-27433.
Ni_3N@C/NF	48	100	1 M KOH seawater	J. Mater. Chem. A. 2021, 9, 13562-13569.
Cu_2S@Ni	54	150	Simulated alkaline seawater	J. Power Sources. 2021, 506, 230235.
Co-Fe_2P	85	25	Simulated alkaline seawater	Appl. Catal. B: Environ. 2021, 297, 120386.
NiMoP/wood	100	26	1 M KOH seawater	Appl. Catal. B: Environ. 2021, 293, 120215.
Co-Ni-P/CP	108	80	Simulated alkaline seawater	J. Mater. Chem. A. 2021, 9, 22248-22253.
NMN-NF	110	50	Simulated alkaline seawater	Electrochimica. Acta. 2021, 390, 138833.
S, P- (Ni, Mo, Fe) OOH/NiMoP/wood	110	10	1 M KOH seawater	Appl. Catal. B: Environ. 2021, 293, 120215.
CoP_x@FeOOH	117	15	1 M KOH seawater	Appl. Catal. B: Environ. 2021, 294, 120256.
Cu_2S	118	12	1 M KOH seawater	Int. J. Hydrogen Energy. 2021, 06, 153.
(Co, Fe)PO_4	137	70	1 M KOH seawater	Nanomaterials. 2021, 11, 2989.

1D-Cu@Co-CoO/Rh	137.7	20	Simulated alkaline seawater	Small. 2021, 17, 2103826.
Ni-SA/NC	139	14	1 M KOH seawater	Adv. Mater. 2021, 33, 2007508.
CoFeZr/NF	140	15	Simulated alkaline seawater	J. Colloid Interface Sci. 2021, 604, 767-775.
$Cu_{0.98}La_{0.02}O$ (CS)	170	2.5	1 M KOH seawater	Appl. Mater. Today. 2021, 24, 101079.
NiFe LDH/FeOOH	180	25	1 M KOH seawater	Inorg. Chem. 2021, 60, 17371-17378.
2D meso-Mo_2C/Mo_2N	197	22	1 M KOH seawater	Angew. Chem. Int. Ed. 2021, 12, 298.
Ni_2P-Fe_2P@NF	220	48	1 M KOH seawater	Adv. Funct. Mater. 2021, 31, 2006484.
PANI-Ni:PANI-Co	335	17	Simulated alkaline seawater	Molecules. 2021, 26, 5926.
C400P350	454	24	Simulated alkaline seawater	J. Power Sources. 2021, 486, 229351.

Supplementary Table 8. Comparison of HER activity measured for R-CoC_2O_4@MXene with other representatives reported HER catalysts using natural seawater as electrolyte.

Sample	Overpotential (mV) 10 mA cm^{-2}	Stability (h)	References
**R-CoC_2O_4@MXene**	**163**	**100**	**This work**
NiCoN|Ni_xP|NiCoN	165	24	ACS. Energy. Lett. 2020, 5, 2681-2689.
Mn-NiO-Ni/Ni-F	170	14	Energy. Environ. Sci. 2018, 11, 1898-1910.
$Cu_{0.98}La_{0.02}O$	170	12	Appl. Mater. Today. 2021, 24, 101079.
GDY/MoO_3	230	120	J. Am. Chem. Soc. 2021, 143, 8720-8730.
Co-FePO/OH	240	10	ACS Appl. Mater. Interfaces. 2021, 13, 53798-53809
Mo_5N_6	257	100	ACS. Nano. 2018, 12, 12761-12769.
Pt@mh-3D MXene	280	250	Adv. Funct. Mater. 2020, 30, 1910028.
Rh/NHCSs	280	13	Green. Chem. 2021, 23, 4551-4559.
NiCoP/NF	287	12	ACS. Appl. Energy. Mater. 2019, 2, 3910-3917.
NiFeP-NS	290	NA	Appl. Catal. B: Environ. 2021, 2022, 120862.
Ti/NiMo	300	14	J. Alloys Compd. 2018, 732, 248-256.
Mn-Ni-S/NF	301	20	J. Mater. Chem. A. 2019, 7, 25628-25640.
$Co_{0.31}Mo_{1.69}C$/MXene/NC	312	225	Adv. Energy Mater. 2019, 9, 1901333.
Rh@N,S-C	320	10	ACS Sustain. Chem. Eng. 2019, 7, 18835.
2D meso-Mo_2C/Mo_2N	341	22	Angew. Chem. Int. Ed. 2021, 12, 298.

NP-MoS_2/CC	345.4	8	Nano. Energy. 2019, 58, 862-869.
Mo_2C-MoP NPC/CFP-800	346	16	Electrochim. Acta. 2018, 281, 710-716.
Ti@Ni$(OH)_2$-NiMoS	371	12	J. Colloid Interface Sci. 2021, 606, 1004-1013.
NF@Mo-$Ni_{0.85}$Se	378	12	Chem. Eng. J. 2021, 422, 130125.
NiCoFeP@NiCoP/NF	400	10	Appl. Surf. Sci. 2019, 489, 519-527.
CoMoP@C	450	10	Energy. Environ. Sci. 2017, 10, 788-798.
NiMo@C_3N_5	486	10	Chem. Eng. J. 2022, 438, 135379.
Fe-Co_2P BNRs	489	100	J. Energy Chem. 2021, 55, 92-101.
Pt/Ni	550	12	Appl. Surf. Sci. 2017, 413, 360-365.
Ni-MoO_2	581	24	ACS Sustain. Chem. Eng. 2021, 9, 13106-13113.

Supplementary References

1. Kibsgaard, J. et al. Designing an improved transition metal phosphide catalyst for hydrogen evolution using experimental and theoretical trends. *Energy Environ. Sci.* **8**, 3022-3029 (2015).
2. Yu, L. et al. Ternary $Ni_{2(1-x)}Mo_{2x}P$ nanowire arrays toward efficient and stable hydrogen evolution electrocatalysis under large-current-density. *Nano Energy* **53**, 492-500 (2018).
3. Benck, J. D., Chen, Z., Kuritzky, L. Y., Forman, A. J. & Jaramillo, T. F. Amorphous molybdenum sulfide catalysts for electrochemical hydrogen production: insights into the origin of their catalytic activity. *ACS Catalysis* **2**, 1916-1923 (2012).
4. Kibsgaard, J., Chen, Z., Reinecke, B. N. & Jaramillo, T. F. Engineering the surface structure of MoS_2 to preferentially expose active edge sites for electrocatalysis. *Nat. Mater.* **11**, 963-969 (2012).
5. Zhou, H. et al. Efficient hydrogen evolution by ternary molybdenum sulfoselenide particles on self-standing porous nickel diselenide foam. *Nat. Commun.* **7**, 12765 (2016).
